# Identification of CD164 as an essential entry receptor for divergent adeno-associated viruses

**DOI:** 10.1073/pnas.2525865123

**Published:** 2026-03-05

**Authors:** Xiujuan Zhang, Donovan Richart, Shane McFarlin, Fang Cheng, Soo Yeun Park, Anwen Zhang-Chen, Richenda McFarlane, Chuan Xiao, Ziying Yan, Jianming Qiu

**Affiliations:** ^a^Department of Microbiology, Molecular Genetics and Immunology, University of Kansas Medical Center, Kansas City, KS 66160; ^b^Division of Pulmonary, Allergy and Critical Care Medicine, Department of Medicine, University of Alabama at Birmingham, Birmingham, AL 35294; ^c^GeneGoCell Inc., San Diego, CA 92127; ^d^Department of Chemistry and Biochemistry, University of Texas at El Paso, El Paso, TX 79968

**Keywords:** adeno-associated virus, CD164, receptor, transduction

## Abstract

Recombinant adeno-associated viruses (rAAVs) are widely used for in vivo gene delivery. While KIAA0319L, known as AAV receptor (AAVR), is essential for the transduction of multiserotype AAVs, it is dispensable for AAV4-related (Clade G) AAVs. We conducted a genome-wide CRISPR/Cas9 screen and identified CD164, a type I transmembrane sialomucin, as an essential entry receptor for Clade G AAVs. Ablation of CD164 expression substantially impaired both entry and transduction of Clade G AAVs. CD164-targeting antibodies and soluble CD164 ectodomain effectively blocked transduction. AAV4 capsids colocalized with CD164 at the plasma membrane and in endosomal compartments. In vitro, CD164 interacted with AAV4 or AAVrh32.33 capsids at high affinity. Importantly, systemic administration of rAAV4 or rAAVrh32.33 in *CD164* knockout (KO) mice resulted in nearly complete loss of transgene expression. These findings establish CD164 as an essential entry receptor for Clade G AAV vectors and uncover a distinct AAVR-independent mechanism of AAV tropism.

## Significance

rAAVs are widely used vectors for human gene therapy, yet the mechanisms governing their entry into host cells remain incompletely understood. While multiple AAV serotypes depend on the broadly expressed AAV receptor (AAVR) for cellular entry, AAV4-related Clade G AAVs transduce cells via an AAVR-independent mechanism. In this study, we identify that CD164, a transmembrane sialomucin located on the cell surface and in endosomal compartments, is essential to the entry and transduction of Clade G AAVs. CD164 directly binds to AAV capsids in vitro and colocalizes with the capsids during cell entry and endosomal trafficking. These findings expand our understanding of AAV–host interaction and reveal a distinct AAVR-independent entry mechanism with implications for vector design and gene therapy.

Recombinant adeno-associated viruses (rAAVs) are widely used gene delivery vectors due to their nonpathogenic nature, broad tissue tropism, and ability to confer stable, long-term transgene expression in diverse cell types and organs. Their relatively low immunogenicity and favorable safety profile have positioned rAAV vectors at the forefront of gene therapy vehicles across a broad spectrum of inherited and acquired disorders (1, 2). To date, seven rAAV-based human gene therapy medicines have been approved by the United States Food and Drug Administration, including Luxturna (AAV2 capsid), Zolgensma (AAV9), Elevidys (AAVrh74), Roctavian (AAV5), Hemgenix (AAV5), Beqvez (AAV8), and Kebilidi (AAV2) (3–8). Among numerous naturally occurring AAV serotypes and variants of engineered capsids, AAV4 stands out for its unique capsid structure, distinct receptor usage, and atypical tissue tropism, distinguishing it from other well-characterized serotypes (9–12). AAV4 also shares an evolutionary lineage with AAVrh32.33, AAV11, AAV12, California sea lion AAV (CslAAV), and bovine AAV (BAAV). These AAVs form a phylogenetically distinct group, collectively referred to as AAV4-related or Clade G AAVs, based on sequence similarities in their large capsid protein VP1 (13–15).

The AAV capsid determines the cell and tissue tropism of each serotype or variant, playing a central role in receptor recognition, cellular entry, and intracellular trafficking (16–18). AAV cell internalization proceeds through two steps: initial attachment to glycan-based receptors, followed by interaction with a cell surface proteinaceous receptor to mediate entry. Both steps are serotype-specific, as capsid structure dictates glycan binding preference and the selection of a proteinaceous receptor. For example, heparan sulfate proteoglycan (HSPG) serves as the attachment receptor for AAV2 (19, 20), AAV3 (21, 22), and AAV13 (23), whereas AAV9 utilizes N-linked galactose for cell surface attachment (24, 25). Sialic acid (SIA), a diverse group of acidic sugars typically found at the terminal ends of glycan chains, serves as a key attachment receptor of various AAVs: N-linked SIA mediates the binding of AAV1 (26), AAV5 (27), and AAV6 (26), whereas AAV4 engages O-linked SIA (28, 29).

Following attachment, AAV employs host machinery for entry and gene delivery, including endocytosis, endosomal escape, intracellular trafficking, and nuclear import. Several host factors have been implicated in these processes (30–33). Among them, the type I transmembrane protein KIAA0319L, also known as AAVR, functions as a proteinaceous receptor for multiserotype AAVs across Clades A-F and H (30, 34), and the G protein–coupled receptor GPR108 is essential for intracellular trafficking of most AAV serotypes, except Clade H AAV5 (31). Notably, AAV4-related Clade G serotypes exhibit a unique entry profile: they do not require AAVR for entry but remain dependent on GPR108 for efficient transduction (31, 35, 36). This divergence suggests that Clade G AAVs utilize an alternative set of host factors for entry, particularly a unidentified proteinaceous receptor. Identification of this receptor is key to understanding the unique transduction biology of Clade G AAVs and to expanding their utility as gene therapy vectors, especially for tissues where conventional rAAV vectors are less effective to target.

To address the limited understanding of AAVR-independent entry mechanisms, we conducted a genome-wide CRISPR/Cas9 screen to identify host factors essential for rAAV4 transduction. Using an optimized screening platform with MRE11-knockout (KO) 293-F cells and a chimeric rAAV2.4 vector, we identified CD164, a transmembrane sialomucin, as a critical determinant of AAV4 capsid entry. We demonstrate that CD164 is required for the transduction of rAAV4 and other Clade G AAVs, acting through direct capsid–receptor interactions and mediating vector internalization and endocytic trafficking. These findings uncover a previously unrecognized AAVR-independent AAV entry mechanism and establish CD164 as a key entry receptor for Clade G AAVs, with broad implications for engineering next-generation gene therapies.

## Results

### A Genome-Wide Screen Using a Lentiviral gRNA Library Identified Host Factors that Restrict AAV4 Capsid-Mediated Transduction in 293-F Cells.

To identify essential host factors required for rAAV4 entry, we performed a genome-wide CRISPR/Cas9 screen using MRE11-KO 293-F cells and a chimeric rAAV2.4 (*SI Appendix*, Fig. S1*A*). The rAAV2.4 vector was engineered by replacing the coding region of AAV4 VP1 N-terminal unique region (VP1u) with that of AAV2, thereby enhancing the transduction of AAV4 while preserving its native tropism (37). The AAV VP1u governs endosomal escape and nuclear localization (38), whereas the remaining coding sequence, shared by VP2 and the major capsid protein VP3, determines capsid structure, receptor binding, and tissue tropism. The 293-F^MRE11-KO^ cell line was created by disruption of the *MRE11* gene, a known restriction factor of AAV transduction (39). These modifications enabled robust rAAV4 transduction, achieving 95% of the cells being transduced (GFP-positive) at a multiplicity of infection (MOI) of 50,000 (50 K) DNase digestion-resistant particles (DRP) per cell (*SI Appendix*, Fig. S1*B*). Using this optimized platform, we performed two rounds of fluorescence-activated cell sorting (FACS) to enrich the cells resistant to rAAV2.4 transduction. The first round yielded ~5% GFP-negative (GFP^−^) cells. After expansion, the cells underwent a second round of sorting, which enriched the GFP^−^ cell population to 98% (*SI Appendix*, Fig. S1*C*). After expansion, gDNA was extracted from the expanded cells. Next-generation sequencing (NGS) of the gDNA samples from the GFP^−^ cells and unsorted cells was carried out, and the data were analyzed using MAGeCK software (Dataset S1). Differential analysis of sorted (GFP^−^) vs. unsorted cells (Sort 2 vs. Sort 0) revealed several significantly enriched gRNAs (*SI Appendix*, Fig. S1*D*). Among the top hits were *TM9SF2*, *ASNA1*, *ST3Gal1*, and *CAMLG*, previously implicated in restricting AAV transduction across various serotypes (32, 33). Notably, *CD164* emerged as a novel and highly enriched gene whose loss conferred resistance to rAAV2.4 transduction, suggesting a previously unrecognized role of CD164 in rAAV4 capsid-mediated transduction.

### CD164 Is Essential for Efficient Transduction of rAAV4 in Cell Lines.

**Fig. 1.**
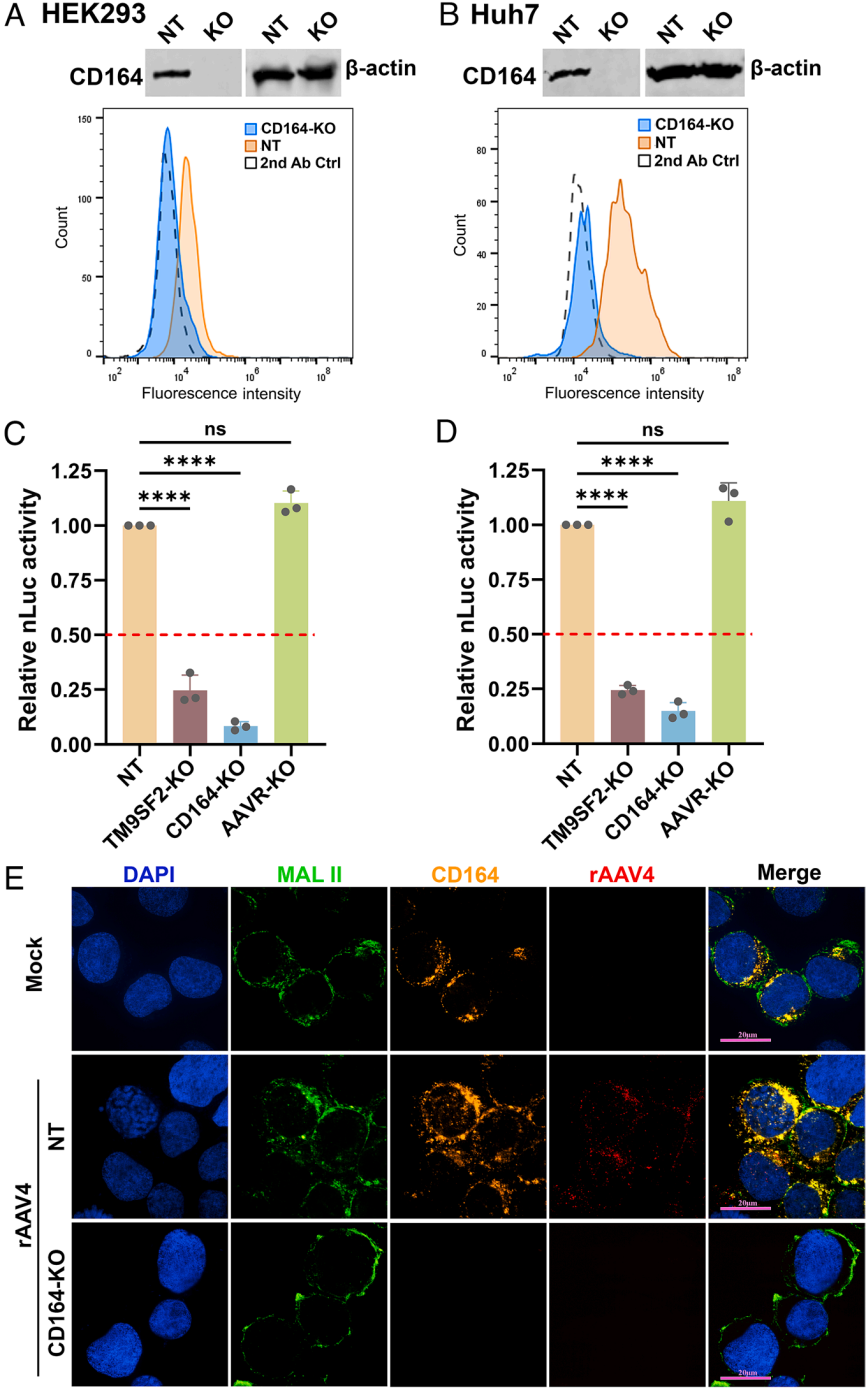
CD164 is essential for efficient transduction of rAAV4 vectors. (*A*) CD164 expression and knockout (KO) in HEK293 cells. Western blotting analysis showed the depletion of CD164 protein in HEK293^CD164-KO^ cells. *β*-actin serves as a loading control. CD164*-*KO or NT control HEK293 cells were stained with *α*-CD164 and Alexa 488-conjugated secondary (2nd) antibodies, followed by flow cytometry. The histograms show the intensity of the Alexa 488 staining on the *x*-axis and the number of cells at each intensity level on the *y*-axis. (*B*) CD164 expression and KO in Huh7 cells. Western blotting analysis showed the depletion of CD164 protein in Huh7^CD164-KO^ cells, and flow cytometry confirmed no expression of CD164 on Huh7^CD164-KO^ cells. (*C* and *D*) Luciferase activities in rAAV4-transduced gene KO cells. HEK293*^CD164^*^KO^ (*C*) or Huh7 *^CD164^* ^KO^ (*D*) cells were transduced with nLuc-expressing rAAV4 at an MOI of 50 K DRP/cell. At 3 dpt, nLuc was measured. Data represent relative transduction efficiency as means ± SD from three replicates, normalized to that of nontargeting (NT) control cells (set to 1.00). The dashed line indicates 50% relative nLuc activity compared to the NT control. (*E*) rAAV4 and CD164 colocalization. NT or CD164-KO HEK293 cells were incubated with rAAV4 at an MOI of 50 K DRP/cell at 4 °C for 2 h, followed by incubation at 37 °C for 1 h. Cells were fixed and stained with biotinylated MAL II lectin, followed by permeabilization and costained with *α*-CD164 and *α*-AAV4 capsid. Images were acquired using a CSU-W1 SoRa spinning disk confocal microscope at 60× magnification, and show AAV4 capsids (red), CD164 (orange), and *α*2-3-linked SIA on plasma membrane (green). Nuclei were stained with DAPI (blue). (Bar, 20 µm.)

CD164, a type I transmembrane sialomucin, is a 197-amino acid cell adhesion molecule that is broadly expressed as a homodimer across human tissues (40). Its extracellular domain (ECD) is known to regulate the proliferation, adhesion, and migration of hematopoietic progenitor cells (41). To investigate its role in AAV transduction, we generated CD164-KO HEK293 cells (Fig. 1*A*) and assessed transduction efficiency using an rAAV4 vector. The results revealed that loss of CD164 significantly reduced transduction by 91.6%, compared to the NT control cells transduced with a nontarget (NT) gRNA-expressing lentivirus, surpassing the reduction observed in TM9SF2-KO cells (Fig. 1*C*). These findings were independently validated in Huh7 cells, where CD164*-*KO likewise resulted in a marked decrease in rAAV4 transduction (Fig. 1 *B* and *D*). We further analyzed the colocalization of AAV4 capsid with CD164 during the early stage of transduction. AAV4 capsids colocalized with CD164 on the cell surface of HEK293 cells, marked by staining of Maackia amurensis lectin (MAL) II at 1 h postentry; however, in CD164-KO cells, AAV4 capsids were nearly undetectable in the cells (Fig. 1*E*).

Taken together, these results demonstrated that CD164 is an essential host factor for efficient transduction by the rAAV4 vector and likely mediates capsid entry.

### Ablation of CD164 Expression Reduces Transduction of rAAV4 in Well-Differentiated Human Large and Small Airway Epithelia.

Given the high transduction of AAV4 in airways (10, 11), we investigated the role of CD164 in rAAV4-mediated transduction of polarized airway epithelia cultures grown at an air–liquid interface (ALI). The human airway ALI model recapitulates the structure and function of the human pseudostratified mucociliary epithelium and is widely used as a model for airway gene transfer (42–44). To assess the impact of CD164, we compared rAAV4 transduction in the ALI cultures derived from both human large- and small-airway epithelial cells lacking CD164.

Using CRISPR/Cas9, we disrupted CD164 expression in CuFi-8 cells, a human large-airway epithelial (HAE) cell line, and in primary human small-airway epithelial cells (HSAE). These cells were subsequently differentiated at ALI to generate HAE-ALI^CD164-KO^ and HSAE-ALI^CD164-KO^ cultures, respectively. Flow cytometry confirmed loss of CD164 expression in both KO cultures (*SI Appendix*, Fig. S2 *A* and *B*), and transepithelial electrical resistance (TEER) measurements exceeded 1,800 Ω·cm^2^, indicating that ablation of CD164 expression did not compromise the epithelial barrier integrity (*SI Appendix*, Fig. S2*C*). We next evaluated the transduction efficiency of rAAV4 in both KO and matched NT control ALI cultures. Under 2 µM doxorubicin (Dox) treatment, a condition commonly used to enhance rAAV transduction in HAE (32), loss of CD164 led to 83.1% and 94.5% reductions in rAAV4 transduction in large- and small-airway derived CD164*-*KO ALI cultures, respectively, compared with their respective NT controls (*SI Appendix*, Fig. S2 *D* and *E*). Notably, even without Dox treatment, rAAV4 efficiently transduced HSAE-ALI, as evidenced by robust GFP expression; however, CD164 loss again resulted in a 94.0% reduction in transduction (*SI Appendix*, Fig. S2 *F* and *G*). In contrast, rAAV4 poorly transduced HAE-ALI cultures without Dox, but CD164 loss still significantly reduced luciferase activity (*SI Appendix*, Fig. S2 *H* and *I*).

Collectively, these differences indicate a CD164-dependent transduction of rAAV4 in both large- and small-airway epithelia. While the Dox treatment enhances the transduction of large-airway epithelium, as observed with other AAV serotypes, small-airway epithelium appears to be more permissive to AAV4 even without Dox treatment.

### CD164 Shares a Common Essential Role for Transduction of AAV4-Related (Clade G) AAVs but Not for Clades A-F or AAV5-Related (Clade H) AAVs.

**Fig. 2.**
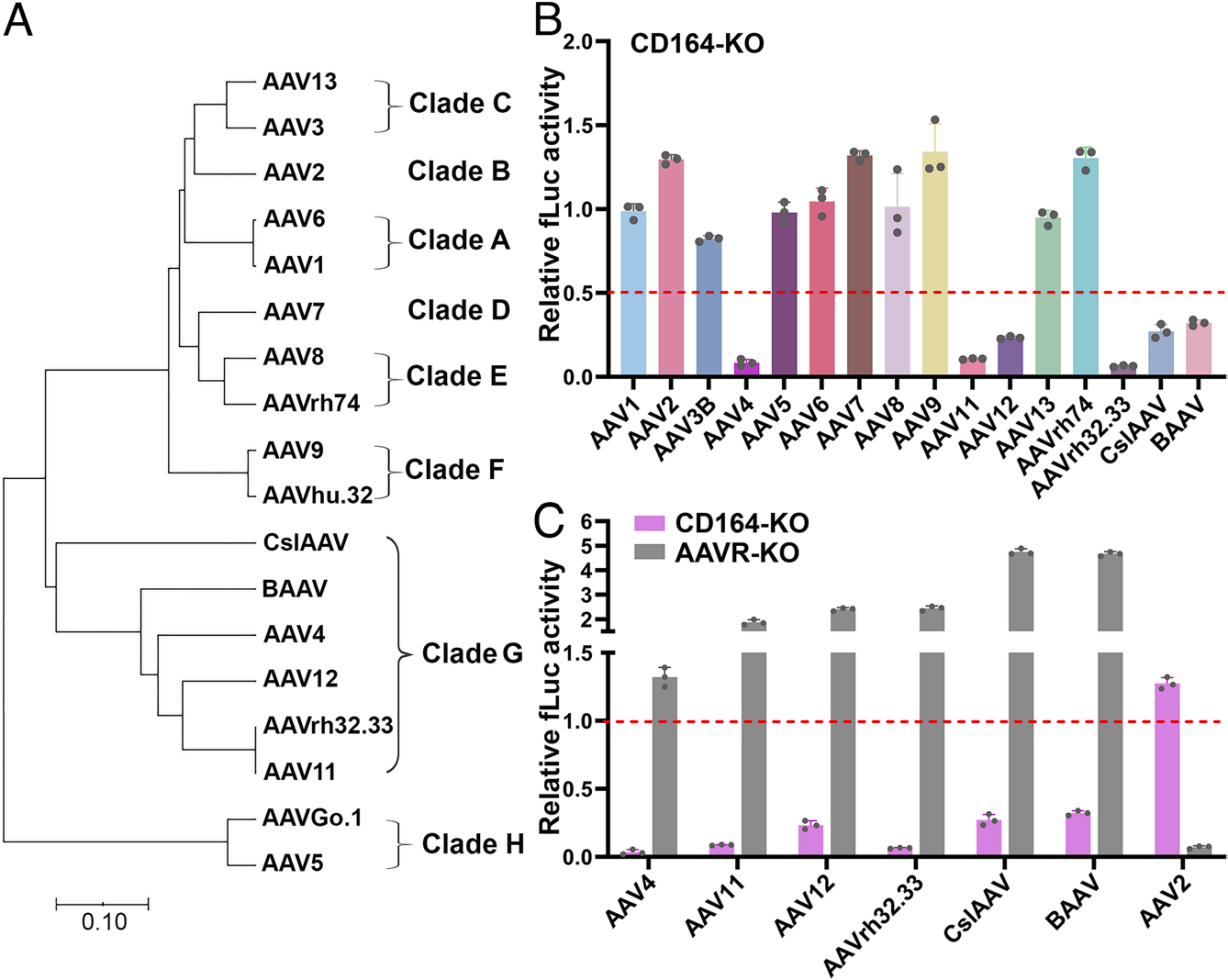
Ablation of CD164 expression significantly diminishes the transduction of Clade G rAAVs in HEK293 cells but increases the transduction of Clade A–F and Clade H rAAVs. (*A*) Phylogenetic analysis of AAV capsid sequences across clades. A phylogenetic tree of AAV VP1 sequences was generated using the MEGA11 program. The tree groups AAVs into eight distinct clades (A–H). The VP1 sequences used are as follows in GenBank: AAV1 (NC_002077), AAV2 (NC_001401), AAV3 (NC_001729), AAV4 (NC_001829), AAV5 (NC_006152), AAV6 (AF028704), AAV7 (NC_006260); AAV8 (NC_006261), AAV9 (AY530579), AAV11 (AY631966), AAV12 (DQ813647); AAV13 (EU285562); AAVhu.32 (AAS99282), AAVrh32.33 (EU368926), BAAV (NC_005889), CslAAV (JN420371), and AAVGo.1 (AY724675). AAVrh74 VP1 sequence is not available at GenBank (47). The scale bar represents 0.10 substitutions per amino acid position. (*B*) rAAV transduction in CD164*-*KO HEK293 cells. HEK293^NT^ and HEK293^CD164-KO^ cells were transduced with various serotypes or variants of rAAV vectors, as indicated, at an MOI of 20 K DRP/cell. (*C*) Clade G AAV transduction in HEK293^AAVR-KO^ and HEK293^CD164-KO^ cells. WT and HEK293^AAVR-KO^ cells, as well as HEK293^NT^ and HEK293^CD164-KO^ cells, were transduced with Clade G rAAV vectors, as indicated, at an MOI of 20 K DRP/cell. At 3 dpt, fLuc activities were measured and normalized to the value in the paired transduced NT control cells (set to 1.0), and are shown with means ± SD from three replicates.

To investigate whether CD164 serves as a general entry factor across multiserotype AAVs, we evaluated the transduction of a diverse panel of rAAV vectors in HEK293 cells. Based on the VP1 sequence phylogeny, these naturally occurring AAVs are classified in Clades A–F, as well as the AAV4-related Clade G and the AAV5-related Clade H (Fig. 2*A*) (13–15, 45, 46). CD164*-*KO selectively impaired transduction by all Clade G AAVs tested, including AAV4, AAV11, AAV12, AAVrh32.33, CslAAV, and BAAV, indicating a shared dependency on CD164 in this clade (Fig. 2*B*). In contrast, *AAVR* KO significantly enhanced transduction by these Clade G AAVs (Fig. 2*C*), consistent with previous observations that Clade G AAVs utilize an AAVR-independent entry pathway.

Taken together, these findings suggest that CD164 is a common and essential host factor for transduction of Clade G rAAV vectors, acting through a mechanism that is distinct and independent of the canonical AAVR pathway.

### CD164 Is a Key Host Factor Mediating Clade G AAV Entry.

**Fig. 3.**
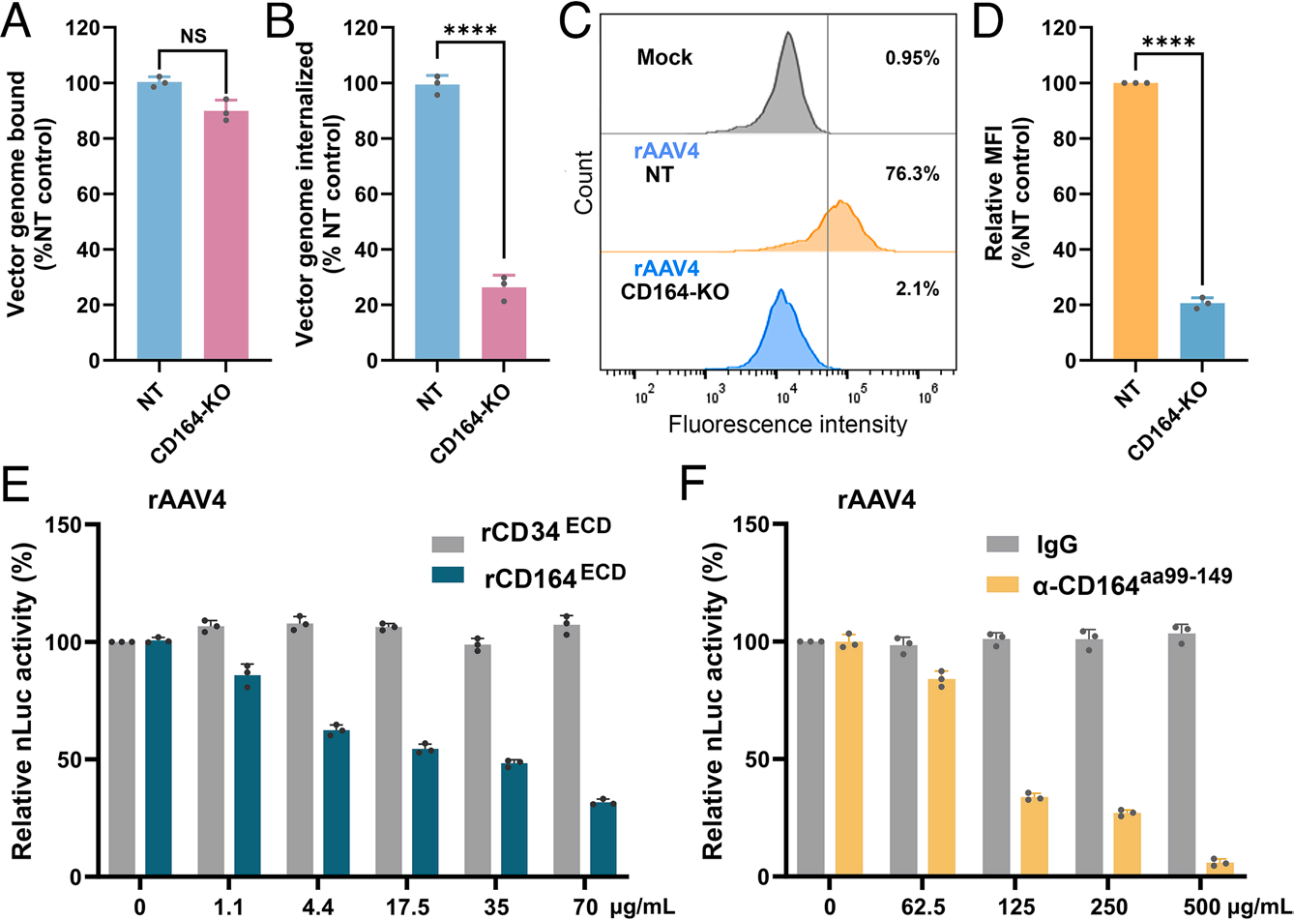
CD164 mediates Clade G AAV vector entry. (*A* and *B*) rAAV4 binding and internalization. NT control and CD164*-*KO HEK293 cells were transduced with rAAV4 at an MOI of 50 K DRP/cell for both vector binding and entry assays. Relative percentages of vector binding (*A*) and internalization (*B*) to the NT control cell group (set as 100% for normalization) are calculated. The data shown are mean ± SD from three replicates. **P < 0.001; ns, no significance. (*C* and *D*) Flow cytometry analysis of AAV4 entry. NT and CD164*-*KO HEK293 cells were transduced with rAAV4 at an MOI of 50 K at 4 °C for 2 h and then at 37 °C for 1 h. The cells were fixed, permeabilized, and stained with *α*-AAV4 followed by Alexa Fluor 647-conjugated secondary antibody. Flow cytometry was performed to quantify internalized AAV4 particles. (*C*) A representative flow cytometry histogram is shown with the percentage of AAV-positive cells in each cell group as indicated. A reference line corresponding to the mock-infected group (gray) is shown. (*D*) Quantification of relative mean fluorescence intensity (MFI) from panel *C*, normalized to the NT group (set as 100%). Relative MFI values are presented as mean ± SD from three independent experiments. ****P < 0.0001. (*E*) Inhibition of transduction by soluble recombinant CD164 ectodomain (ECD). rAAV4 at an MOI of 50 K was preincubated with various concentrations (0 to 70 µg/mL) of recombinant CD164 ECD (rCD164^ECD^) or CD34 ECD (rCD34^ECD^) in serum-free medium at 37 °C for 30 min. The vector–protein mixtures were then added to HEK293 cells and incubated for 2 h, followed by the addition of fresh media. At 3 dpt, luciferase activity was measured and normalized to the value in mock-treated cells (set up as 100% for relative nLuc activity). The data shown are mean ± SD from three replicates. (*F*) Anti-CD164 antibody block of transduction. HEK293 cells were incubated with an α-CD164 (aa99–149) or an IgG control at indicated concentrations at 4 °C for 1.5 h before rAAV4 transduction at an MOI of 50 K. At 3 dpt, luciferase activity was measured and normalized to the value in the mock-treated cells. The data shown are mean ± SD from three replicates.

To assess the role of CD164 during the entry of Clade G AAVs, we analyzed rAAV4 binding and internalization in HEK293 cells. We quantified vector genomes associated with the cell surface (bound) and those internalized into the cells after infection, using qPCR. While the cell surface-bound vector genomes were comparable between CD164*-*KO and NT control cells, internalized vector genomes were significantly reduced by 73.7% in CD164*-*KO cells (Fig. 3 *A* and *B*). Further flow cytometry analysis for intracellular capsids confirmed a 79.4% reduction in capsid uptake in CD164*-*KO cells (Fig. 3 *C* and *D*). These results demonstrated that CD164-KO is dispensable for the initial attachment of AAV4 to cells but indispensable for its internalization, highlighting its key role in facilitating Clade G AAV uptake.

To determine whether CD164 mediates viral uptake through its ECD, we tested the ability of a recombinant ECD of CD164 (rCD164^ECD^) and α-CD164^aa99–149^, a polyclonal antibody targeting amino acid (aa)99–149, as diagrammed in *SI Appendix*, Fig. S3*A*, in inhibition of rAAV4 transduction. Both reagents significantly inhibited rAAV4 transduction in a dose-dependent manner, but not recombinant CD34 ectodomain (rCD34^ECD^) derived from another O-glycosylated sialomucin (48) (Fig. 3 *E* and *F*). In addition, two monoclonal antibodies (clones N6B6 and 67D2), which recognize conformational epitopes within the disulfide bridge domain of CD164 (49) (*SI Appendix*, Fig. S3*A*), also suppressed rAAV4 transduction in a dose-dependent manner (*SI Appendix*, Fig. S3 *B* and *C*). We extended these findings to rAAVrh32.33, another Clade G AAV. rCD164^ECD^, α-CD164^aa99–149^ polyclonal, and 67D2 monoclonal antibodies all inhibited rAAVrh32.33 transduction in a dose-dependent manner (*SI Appendix*, Fig. S3 *D–F*), supporting a conserved mechanism across Clade G serotypes.

Taken together, these results provide strong evidence that CD164 facilitates entry of Clade G AAVs through an interaction involving its ECD, reinforcing its role as a key entry receptor for Clade G phylogenetic group AAVs.

### The MD1 and MD2 Domains of CD164 Are Essential for rAAV4 Transduction.

**Fig. 4.**
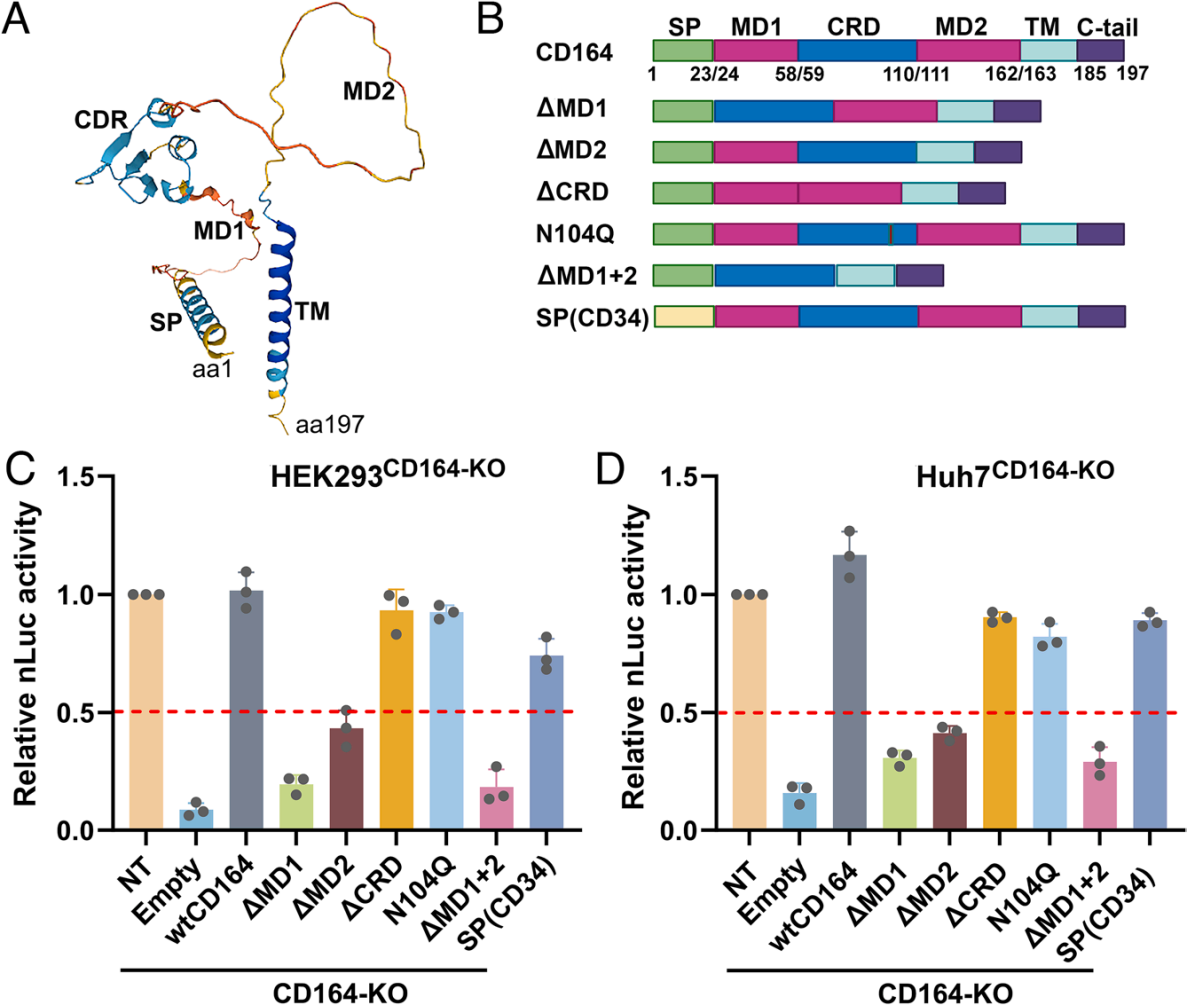
The mucin domains of CD164 are critical for rAAV4 transduction. (*A*) Predicted structure of CD164. The structure of human CD164 is predicted using AlphaFold3, and is shown with MD1, CRD, and MD2 domains. (*B*) CD164 mutants used in complementation studies. WT CD164 and six truncation and one-point mutants are schematically diagrammed. (*C* and *D*) rAAV4 transduction. CD164-KO HEK293 (*C*) and Huh-7 (*D*) cells were transduced with lentiviral empty or the vectors expressing full-length WT or mutant CD164, followed by selection with blasticidin. Cells were then transduced with nLuc expressing rAAV4 at an MOI of 50 K DRP/cell. At 3 dpt, nLuc activity was measured. Data represent fold change relative to the value in NT control cells (set as 1.0), and are shown with mean ± SD from three replicates.

CD164 comprises a signal peptide (SP) at its N-terminus, which is crucial for its translocation to the cell membrane, a transmembrane domain (TM), and the ECD that contains two O-glycan–rich mucin-like domains (MD1 and MD2) and one cysteine-rich domain (CRD) (Fig. 4 *A* and *B*) (41). MD1 and MD2 are heavily O-glycosylated, whereas CRD contains four N-linked glycosylation sites. To define the functional regions of CD164 required for rAAV4 transduction, we generated a series of domain-deletion mutants. We also introduced a point mutation (N104Q) at an N-linked glycosylation site previously known to be critical for lymphocytic choriomeningitis virus (LCMV) entry (50, 51), and replaced the native signaling peptide with that of CD34 [SP(CD34)] (51). Wild-type (WT) CD164 (wtCD164) and these mutants were expressed in CD164*-*KO HEK293 and Huh7 cells, respectively, which exhibited similar expression levels, as determined by both western blotting and flow cytometry (*SI Appendix*, Fig. S4 *A–D*), to evaluate their ability to rescue rAAV4 transduction.

CD164*-*KO cells that expressed a CD164 mutant ∆MD1 or ∆MD2 exhibited over 50% reduction, while the cells expressing ∆MD1+2 resulted in 83.4% and 87.6% decrease in rAAV4 transduction in HEK293 and Huh7 cells, respectively, compared to those expressing wtCD164 (Fig. 4 *C* and *D*). Expression of other mutants in the CD164*-*KO cells exhibited a compensatory function comparable to the wtCD164 in rescuing rAAV4 transduction (Fig. 4 *C* and *D*).

AAV4 uses O-linked sialoglycans on the cell surface for primary attachment (28, 29). Since the ECD of CD164 is heavily O-glycosylated (41), we examined whether CD164*-*KO and deletion of its individual domains altered the global O-glycosylation on the cell surface, using Jacalin lectin staining. The results revealed no substantial differences in Jacalin staining across the tested groups: NT control, CD164-KO, wt or mutant CD164-expressing CD164-KO cells (*SI Appendix*, Fig. S4 *E* and *F*). Similarly, wheat germ agglutinin (WGA) lectin staining, which detects both O- and N-glycans, showed no differences across groups (*SI Appendix*, Fig. S4 *G* and *H*).

Together, these findings suggest that the MD1 and MD2 domains of CD164 are functionally required for rAAV4 transduction. Although the MD1 and MD2 domains are highly O-glycosylated, their deletion did not affect the global O-glycosylation on the cell surface but significantly decreased rAAV4 vector transduction, supporting that CD164-KO does not affect rAAV4 binding to the cell surface.

### Clade G AAVs Bind Directly to CD164 with High Affinity.

**Fig. 5.**
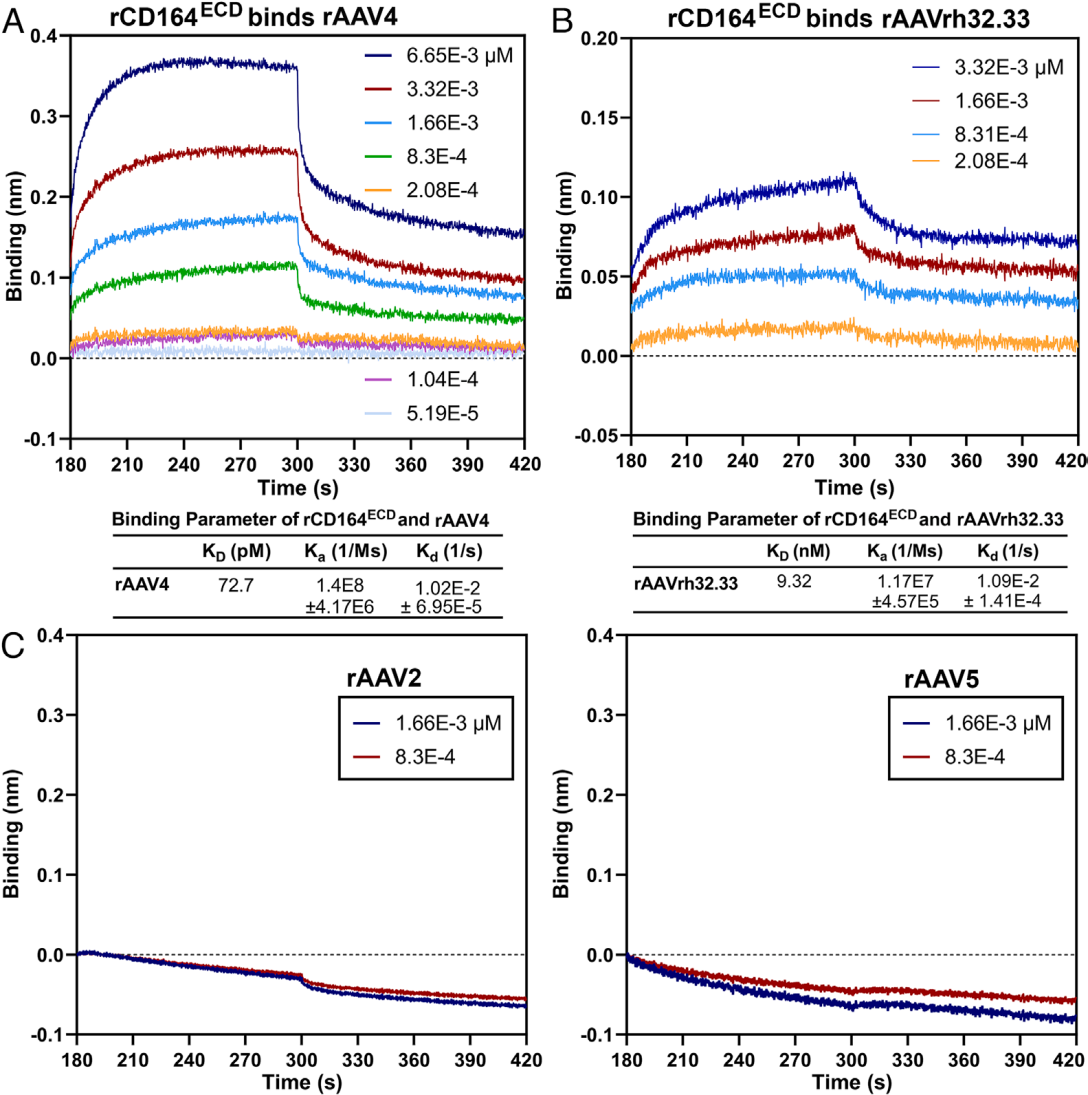
CD164 binds AAV4 and AAVrh32.33 capsids with high affinity measured by biolayer interferometry (BLI). Binding kinetics of His-tagged rCD164^ECD^ on Ni-NTA biosensors to rAAV4 (*A*), rAAVrh32.33 (*B*), rAAV2, and rAAV5 (*C*) was determined, at increasing concentrations as indicated, on an Octet RED96e. The binding of rCD164^ECD^ with rAAV4 (*A*) or rAAVrh32.33 (*B*) shows a dose-dependent increase in binding signal (nm shift) during the association phase (180 to 300 s), followed by a dissociation phase (300 to 420 s). An equilibrium dissociation constant (*K_D_*) value represents the ratio of dissociation [*k_d_* (1/s)] and association [*k_a_* (1/Ms)] computed from the real-time binding curves of the protein with rAAV. The values are shown with means ± SD. (*C*) No detectable binding of rCD164^ECD^ to rAAV2 or rAAV5 at the indicated concentrations was observed.

To determine whether Clade G AAVs directly interact with CD164, we performed biolayer interferometry (BLI) assays using a glycosylated rCD164^ECD^ purified from HEK293 cells (*SI Appendix*, Fig. S5*A*). Both AAV4 and AAVrh32.33 capsids exhibited strong binding to rCD164^ECD^, with equilibrium dissociation constant (*K_D_*) values of 72.7 pM and 9.32 nM, respectively (Fig. 5 *A* and *B*), indicating high affinity interactions. In contrast, AAV2 and AAV5 capsids showed no binding (Fig. 5*C*), confirming that the CD164 interaction is specific to Clade G AAV capsids.

To assess whether the glycosylation on CD164 contributes to this interaction, we compared the AAV-binding properties of CD164 with CD34, using BLI assays. rCD34^ECD^ bound to AAV4 or AAVrh32.33 capsid in a dose-dependent manner, but quickly dissociated from the capsids to the background level (*SI Appendix*, Fig. S5 *B* and *C*), suggesting a nonspecific binding. Furthermore, rCD34^ECD^ failed to inhibit rAAV4 or rAAVrh32.33 transduction in a competition assay (Fig. 3*E* and *SI Appendix*, Fig. S3*D*), indicating that glycosylation alone is insufficient for AAV4 binding. We also evaluated a nonglycosylated version of the CD164 ECD expressed in *Escherichia coli* (rCD164^ECD–E^), visualized as a single band at ~24 kDa on a denatured gel (*SI Appendix*, Fig. S5*A*). It remained bound to AAV4 capsids, albeit with moderately reduced affinity, compared to the glycosylated form (*SI Appendix*, Fig. S5*D*).

Taken together, these findings demonstrate that Clade G AAVs directly bind the CD164 protein backbone with high specificity and affinity. While glycosylation is not essential for the interaction, it contributes to enhancing binding affinity.

### CD164 Facilitates Clade G AAV Internalization and Trafficking through the Endosomal Pathway.

**Fig. 6.**
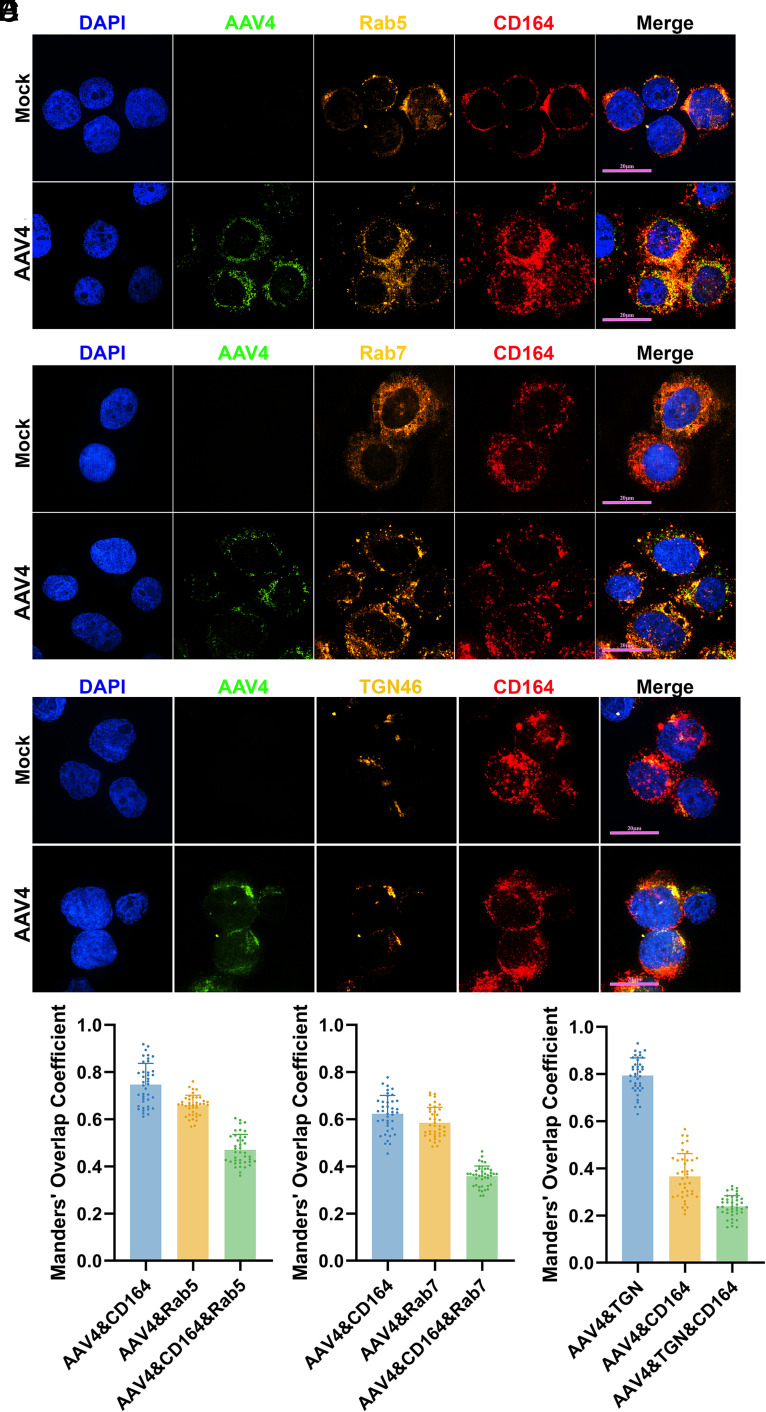
AAV4 colocalizes with CD164 in endosomes and the *trans*-Golgi network. (*A*–*C*) Colocalization of AAV4 (green) with CD164 (red) and subcellular compartment markers (orange). HEK293 cells were transduced with rAAV4 at an MOI of 50 K DRP/cell. (*A*) AAV4 colocalizes with Rab5 and CD164. At 4 hpt, cells are collected and stained to show rAAV4 particles (green), Rab5 (orange), and CD164 (red) in HEK293 cells. (*B*) AAV4 colocalizes with Rab7 and CD164. At 6 hpt, cells are collected and stained to show rAAV4 particles (green) Rab7 (orange) and CD164 (red) in HEK293 cells. (*C*) AAV4 colocalizes with TGN46 and CD164. At 8 hpt, cells are collected and stained to show rAAV4 particles (green), TGN46 (orange), and CD164 (red) in HEK293 cells. Mock-treated cells serve as negative controls in all panels. Nuclei were stained with DAPI (blue). Images were acquired using a CSU-W1 SoRa spinning disk confocal microscope at 60× magnification. (Bar, 20 µm.) (*D*–*F*) Quantification of colocalization signals. Manders’ Overlap Coefficient was calculated for AAV4 colocalization with the indicated markers in single cells (*n* = 40 per group) using ImageJ (Fiji). Each dot represents one cell; bars show mean ± SD.

To investigate the role of CD164 in the intracellular trafficking of Clade G AAV, we tracked AAV4 localization at various time points posttransduction in HEK293 cells. At 4 hpt, rAAV4 capsids showed strong colocalization with CD164 (74.7%), with 47.1% of these signals colocalized in the early endosome marked by Rab5 (Fig. 6 *A* and *D*). By 6 hpt, 35.7% of the CD164-associated capsids were detected in the late endosome marked by Rab7 (Fig. 6 *B* and *E*). At 8 hpt, rAAV4 capsids accumulated in the *trans*-Golgi network (TGN), with 79.4% colocalization with TGN46 (Fig. 6 *C* and *F*). Notably, at this stage, CD164 was mostly dissociated from the capsids. Moreover, expression of ∆CRD mutant exhibited similar colocalizations with AAV4, as well as with Rab5, Rab7, and TGN46 at 4, 6, and 8 hpt in CD164-KO HEK293 cells; but the ∆MD1+2 mutant expression failed to internalize AAV4 capsids (*SI Appendix*, Fig. S6). These findings suggest that CD164 plays a key role in mediating vector endocytosis and intracellular trafficking from early to late endosomes with progressive disassociation from the vector as it transits toward the TGN.

Since endosomal maturation is associated with a progressive decrease in pH, from ~6.5 in early endosomes to ~5.5 in late endosomes (52), we next assessed the pH dependence of rAAV4 binding to rCD164^ECD^ using BLI. The results revealed that rAAV4 exhibited the strongest binding to CD164 at pH 6.5 to 7.0 (*SI Appendix*, Fig. S5*E*), reflecting the extracellular and early endosomal environments. Binding was markedly reduced at lower pH value (5.5 to 6.0) (*SI Appendix*, Fig. S5*E*), consistent with late endosomal conditions. These results support a model in which CD164 binds AAV4 capsids at the cell surface and in the early endosomes, but gradually dissociates from the capsid as it traffics through increasingly acidic compartments (late endosomes). A similar trafficking pattern was observed with rAAVrh32.33, which also strongly colocalized with CD164 and showed moderate localization with Rab5^+^ or Rab7^+^ endosomes (*SI Appendix*, Fig. S7).

Collectively, these findings support a model in which CD164 mediates Clade G AAV internalization and guides capsid trafficking from the early endosome to the late endosome and ultimately to the TGN, with VP1u externalization during endosomal maturation.

### CD164 Overexpression Enhances Clade G rAAV Transduction.

To further evaluate the functional role of CD164 in promoting AAV transduction, we assessed whether its overexpression could enhance transduction efficiency. Human CD164 (hCD164) and mouse CD164 (mCD164) were overexpressed in cell lines derived from different species, including NIH3T3 (mouse fibroblast), COS7 (monkey kidney fibroblast), and HeLa (human cervical cancer cells) (*SI Appendix*, Fig. S8*A*). Overexpression of either hCD164 or mCD164 led to a 1.65 to 3.97-fold increase in rAAV4 transduction across tested cell lines (*SI Appendix*, Fig. S8*B*). Notably, the enhancement effect was even more pronounced for rAAVrh32.33, and it displayed a species-dependent trend: mCD164 overexpression in mouse-derived NIH3T3 cells resulted in an 11.76-fold increase, whereas hCD164 overexpression in HeLa cells increased transduction by 17.64-fold (*SI Appendix*, Fig. S8*C*). These results demonstrate that CD164 expression level is a significant determinant of Clade G AAV vector transduction across cell types of diverse species.

### CD164 Is Essential for In Vivo Gene Delivery by Clade G AAV Vector.

**Fig. 7.**
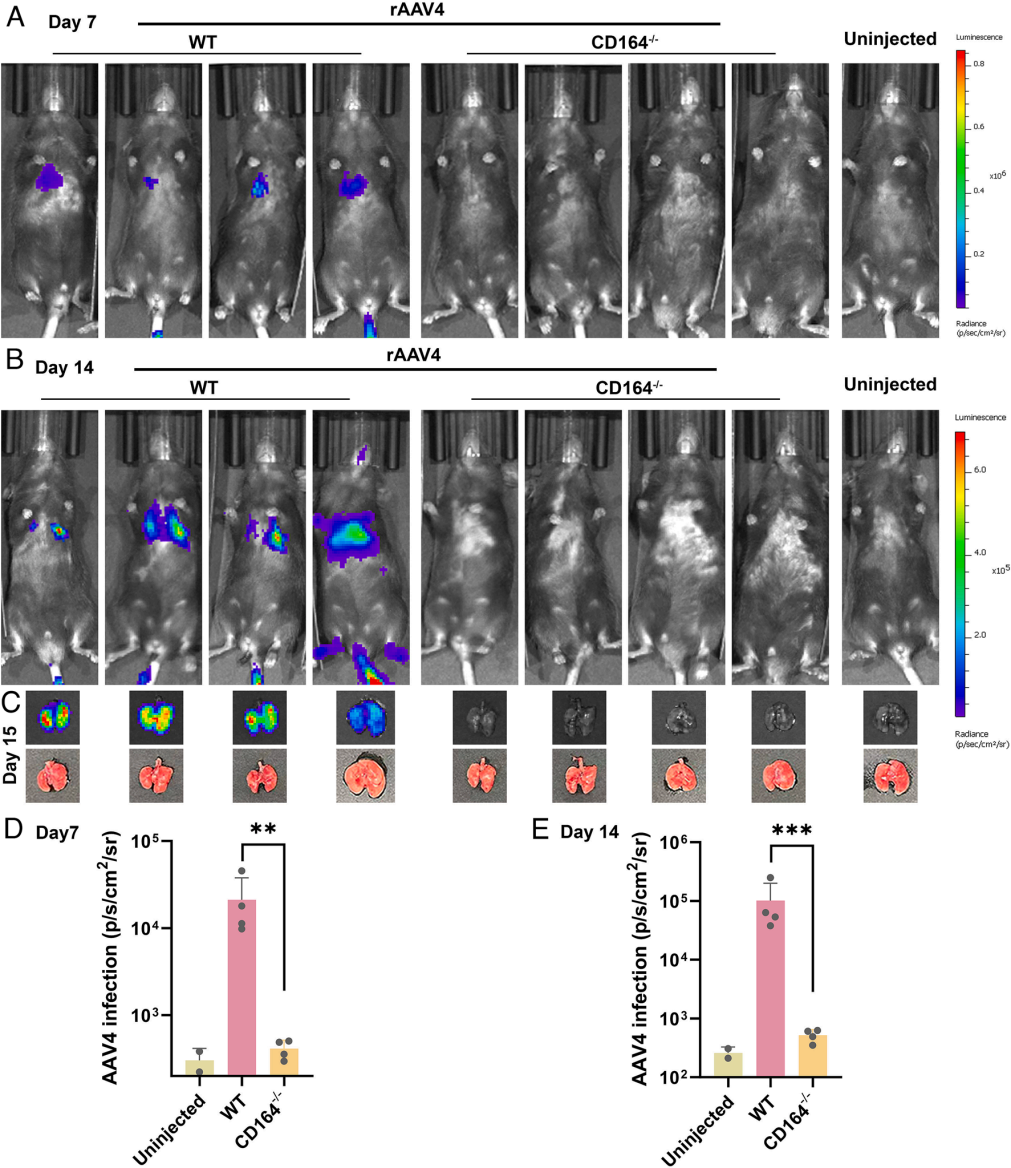
rAAV4 efficiently transduces the lungs of WT mice but not *CD164* KO mice. (*A*–*C*) In vivo and ex vivo bioluminescence imaging. WT (CD164^+/+^) and *CD164* KO (CD164^−/−^) C57BL/6JGpt mice (*n* = 4) were intravenously administrated with fLuc-expressing rAAV4 at 5 × 10^11^ DRP/mouse. (*A* and *B*) In vivo imaging. IVIS. At 7 d (*A*) or 14 d (*B*) postinjection, mice were intraperitoneally injected with D-luciferin and imaged under an IVIS Spectrum system. One representative animal from the uninjected control group (*n* = 2) is shown on the far right of each panel. (*C*) Ex vivo imaging. At 15-d postinjection, mice were intraperitoneally injected with D-luciferin. The mice were euthanized under the University of Kansas Medical Center IACUC-approved Protocol (#24-12-423), and the lungs were harvested for ex vivo bioluminescence imaging. Shown are excised images of lungs corresponding to the mice in panels (*A* and *B*) with overlaid bioluminescence, alongside corresponding brightfield images of the lungs. Pseudocolored images represent luciferase activity (photons/sec/cm^2^/sr) with radiance scales shown on the right. (*D* and *E*) Quantification of whole-body bioluminescence signals. The in vivo imaging signals of the mouse taken at 7-d (*D*) and 14-d (*E*) postinjection were quantified. The mean bioluminescence signal of the uninjected control group represents the background. Each dot represents an individual mouse. Bars show mean ± SD. Statistical significance was determined by one-way ANOVA with multiple comparisons. ***P < 0.001; ns, not significance.

To assess the in vivo relevance of CD164, we generated CD164 KO (CD164^−/−^) mice, which displayed no overt developmental or physiological abnormalities. WT (CD164^+/+^) and homozygous *CD164* KO (CD164^−/−^) C57BL/6Gpt mice (representative genotypes shown in *SI Appendix*, Fig. S9) were administered rAAV4(CMV-GFP-T2A-fLuc) via tail-vein injection. Luciferase expression, used as a readout of transgene delivery, was quantified by in vivo bioluminescence imaging system (IVIS) at 7-, and 14-d postinjection (Fig. 7 *A* and *B*). In WT mice, systemic administration of rAAV4 led to strongly lung-localized bioluminescent signals, whereas little to no signals were detected in CD164^−/−^ mice, at either time point (Fig. 7 *D* and *E*). Consistently, ex vivo bioluminescence imaging of the lungs harvested at 15-d postinjection confirmed robust luciferase expression in WT mice, but not in CD164^−/−^ mice (Fig. 7*C*).

A similar CD164-dependent transduction pattern was observed in vivo with rAAVrh32.33. WT and CD164^−/−^ mice were systematically administrated rAAVrh32.33(CMV-GFP-T2A-fLuc) or as a control, rAAV5(CMV-GFP-T2A-fLuc). In WT mice, administration of rAAVrh32.33 led to strong bioluminescent signals at 6-, 14-, and 21-d postinjection, whereas CD164^−/−^ mice showed no detectable signals above background (the level defined by uninjected animals) (*SI Appendix*, Figs. S10*A* and S11), indicating a nearly complete loss of rAAVrh32.33-mediated transduction. In contrast, rAAV5-mediated luciferase expression was robust and comparable between WT and CD164^−/−^ mice. Quantification of bioluminescent signals confirmed a significant reduction in rAAVrh32.33-mediated transduction in CD164^−/−^ mice at all time points, compared to WT mice (*SI Appendix*, Fig. S10*B*). In contrast, rAAV5 transduction was undistinguished between WT and CD164^−/−^ mice at all time points (*SI Appendix*, Fig. S10*C*). This is consistent with its known CD164-independent and AAVR-dependent entry pathway in cells (Fig. 2 *B* and *C*).

Together, these results provide direct in vivo evidence that CD164 is indispensable for in vivo transduction by Clade G AAV vectors, rAAV4 and rAAVrh32.33, but it is not required for other rAAV vectors like rAAV5 that rely on AAVR for cellular entry.

## Discussion

The transduction of Clade G AAVs, including AAV4, AAVrh32.33, AAV11, AAV12, BAAV, and CslAAV, does not require AAVR, the multiserotype AAV receptor (AAVR) used by Clade A-F and H AAVs (12, 30, 34, 53,54, 55). In this study, we conducted a genome-wide CRISPR/Cas9 screen to identify host factors essential for AAV4 capsid-mediated transduction. Using a GFP-expressing chimeric rAAV2.4 vector bearing AAV2 VP1u in AAV-sensitized MRE11-KO 293-F cells, we identified the type I transmembrane sialomucin CD164 as a bona fide proteinaceous receptor for cell entry of AAV4 and the other AAVR-independent Clade G AAVs.

CD164 is predominantly localized to the plasma membrane and endosomal compartments, and is known to regulate hematopoietic stem cell adhesion, epithelial differentiation, and endosomal trafficking (41). It has also been implicated in the cellular entry of LCMV, where it facilitates viral endocytic trafficking via interaction with viral glycoproteins (50, 51). Our findings expand the role of CD164 in mediating the entry and intracellular trafficking of AAVR-independent Clade G AAVs. Genetic ablation of CD164 significantly reduced the transduction of Clade G AAVs across multiple human cell types and tissues, including polarized human airway epithelia, as well as in vivo in mice. This reduction in transduction reflects impaired vector entry rather than decreased cell surface binding. Either soluble CD164 ectodomain or antibodies targeting it inhibited the transduction of Clade G AAVs. In vitro biochemical analyses revealed that the CD164 ectodomain binds to Clade G AAV capsids with very high affinity, exhibiting a *K_D_* in the sub- to nanomolar range, consistent with high affinity receptor–ligand interactions. Confocal microscopy demonstrates colocalization of Clade G AAV capsids with CD164 at both the plasma membrane and endosomal compartments, but to a much lesser extent in the TGN.

CD164 contains a carboxyl-terminal NYxxL motif, which mediates its trafficking to the endolysosomal compartments (56), and facilitates AAV intracellular transport through the endocytic network. In vitro binding assays further revealed that the interaction between CD164 and Clade G AAV capsids is pH-sensitive, with optimal binding at pH 6.5 to 7.0, conditions corresponding to plasma membrane and early endosomes. These findings support a model, in which Clade G AAVs bind to CD164 on the cell surface and enter cells via endocytosis as an AAV–CD164 complex. In early endosomes, AAV remains associated with CD164; however, as the endosomal environment acidifies, it progressively dissociates from CD164 and externalizes its VP1u during endosomal maturation, facilitating escape from the endosome and allowing it to traffic to the TGN (*SI Appendix*, Fig. S12). All these lines of evidence support CD164 being a critical entry receptor for Clade G AAVs, functioning through high-affinity binding and pH-dependent dissociation during endocytosis and endosomal trafficking.

The requirement for CD164 in AAV transduction is not limited to AAV4 and AAVrh32.33 vectors but extends to multiple serotypes within Clade G AAVs. In contrast, transduction by canonical AAV Clades A–F and H remains unaffected by ablation of CD164 expression. Notably, both rAAV4 and rAAVrh32.33 fail to transduce CD164^−/−^ mice, whereas rAAV5 transduces them as efficiently as WT mice. This differential transduction profile highlights that CD164 serves as a critical proteinaceous receptor specifically for Clade G AAVs, which share a high sequence homology in their VP1 capsid protein, likely reflecting conserved structural features that confer CD164 engagement. Indeed, AAV4, AAVrh32.33, AAV11, and AAV12 exhibit >90% structural identity in their capsids (12, 14, 57), and importantly, these AAV serotypes also transduce cells independently of AAVR (35). Recently, carboxypeptidase D (CPD) was identified as an AAVR-independent alternate receptor for AAV11 and AAV12 (58). Unlike CD164, CPD also facilitates AAV8 transduction in AAVR-KO cells, and its overexpression enhances rAAV8 transduction, even though the AAV8 capsid shares a very low similarity with that of AAV11 and AAV12, and can use AAVR for transduction (14). In this context, CD164 plays a more exclusive role in the transduction of Clade G AAVs. Collectively, the phylogenetic clustering, structural similarity, and AAVR independence of these capsids support a unifying model in which Clade G AAVs rely on CD164 for cellular entry and subsequently intracellular trafficking.

Clade G AAVs, including AAV4, AAVrh32.33, CslAAV, BAAV, and AAV12, have been shown to rely on cellular 2,3-O-linked sialoglycans as primary attachment receptors for transduction (28, 29, 59, 60). An in vivo study also confirmed that the O-linked sialoglycans are essential for rAAV4 transduction (29). Notably, CD164 is a glycoprotein with one N-glycosylated CRD and two O-glycosylated mucin-like domains, MD1 and MD2. While the CRD mediates LCMV endocytosis (51), it does not contribute to AAV4 entry and transduction. Instead, the MD1 and MD2 domains are essential, as indicated by the deletion of both domains which results in a drastic reduction of rAAV4 entry and transduction. Although the MD1 and MD2 domains are extensively O-glycosylated and have been implicated in ligand recognition and protein–protein interactions (49), neither CD164*-*KO nor domain deletion significantly alters the global O-glycosylation level on the cell surface.

In addition, a recombinant CD164 ECD expressed in *E. coli*, which lacks posttranslational glycosylation (*SI Appendix*, Fig. S5*A*), retained high affinity to both AAV4 and AAVrh32.33 capsids. In contrast, CD34, another type I transmembrane sialomucin (*SI Appendix*, Fig. S5*A*), neither bound AAV4 or AAVrh32.33 capsids in vitro nor competitively inhibited rAAV4 transduction (*SI Appendix*, Fig. S5 *B* and *C*). These lines of evidence suggest that the protein backbone of CD164, rather than its O-sialylation, is the primary determinant for capsid interaction. This distinguishes CD164 from general O-glycan–bearing attachment factors, reinforcing its identity as a proteinaceous entry receptor, in which specific amino acid sequences or structural epitopes within MD1 and MD2 mediate AAV binding and/or induce conformational changes necessary for high affinity interaction. While the sialoglycan moieties of CD164 are dispensable for the attachment of AAV4 to the cell surface, they nonetheless enhance the specific protein–capsid contacts and may play a role in postentry steps, such as the pH-dependent dissociation of the receptor–capsid complex during endosome maturation. Structural studies will ultimately be required to precisely define how AAV engages CD164.

From a translational perspective, these findings have important implications for gene therapy. AAV4, which exhibits unique tropism in retinal pigmented epithelium, has been clinically evaluated in ocular gene therapy, particularly for Leber’s congenital amaurosis (61). Beyond the eye, Clade G AAV vectors have been explored for gene delivery to lung epithelial and progenitor stem cells, cardiopulmonary system, neural circuits, and salivary glands (29, 62–66). AAVrh32.33 has been explored for vaccine development due to its unique tropism and limited preexisting immunity (67). Recent research has also revealed a liver-detargeted profile, but a strong preference for AAV4 to transduce vascular endothelial cells and pancreatic beta cells in mice (9). We confirmed that rAAV4 exhibits high tropism for both mouse lungs and differentiated human small airway epithelia (11, 68), and that CD164 is essential for AAV4 transduction, underscoring the potential role as a determinant of tissue tropism and a modulator of gene transfer efficiency, particularly in small airways. CD164 is broadly expressed in human cells and tissues, including airway epithelial cells, gastrointestinal mucosa, endothelial cells, lymphoid tissues, and hematopoietic cells (69). In future applications, CD164 expression profiles in target tissues could guide vector selection or engineering. Moreover, modulation of CD164 levels, either by enhancing expression or transiently blocking its function, may offer a strategy to fine-tune vector targeting small airways of the lungs, for example, in cystic fibrosis gene therapy, and minimize off-target transduction in systemic vector delivery.

In summary, our results establish that Clade G AAV capsids directly interact with CD164 at high affinity in a pH-dependent manner, which supports a model in which CD164 mediates AAV endocytosis and endosomal trafficking from the plasma membrane to and through the endosome network to the TGN. The retention of binding by the nonglycosylated CD164, combined with the inability of CD34, another cell surface sialomucin molecule, to substitute for CD164, further supports the conclusion that CD164 functions as a specific proteinaceous receptor, rather than a generic glycan-presenting surface molecule for Clade G AAV entry. These findings add a layer to the understanding of AAV–host interactions and provide a molecular basis for optimizing the design and application of Clade G AAV-based gene deliveries.

## Materials and Methods

### Cell Lines and Airway Epithelial Cultures.

Cell lines: HEK293 cells (#CRL-1573, ATCC), FreeStyle 293-F cells (#R79007293F, ThermoFisher, Waltham, MA). NIH3T3 (#CRL-1658, ATCC), COS7 (#CRL-1651, ATCC), HeLa (#CCL-2, ATCC), and Huh7 cells (CVCL_0336). Human tracheal (large) airway epithelial cells and small-airway epithelial cells were polarized at an ALI to generate human large- and small-airway epithelium (HAE and HSAE). Details for all cell lines, cultures, and differentiation procedures can be found in *SI Appendix*.

### Plasmid Constructs, Transfection, AAV and Lentivirus Vector Production, and CRISPR/Cas9-Based Gene KO.

Methods of plasmid construction, transfection, vector production, as well as gene KO can be found in *SI Appendix*.

### gRNA Library and Genome-Wide CRISPR/Cas9 Screen, NGS, and Bioinformatics Analysis.

The Brunello lentiCRISPR gRNA library (#73178-LV, Addgene) was used for the genome-wide CRISPR/Cas9 screen. A detailed method can be found in *SI Appendix*.

### rAAV Vector Cell Transduction, Binding, and Entry Assays.

We carried out the rAAV transduction of cells, vector binding, and entry assays as previously published (33). Detailed methods can be found in *SI Appendix*.

### Immunofluorescent Confocal Microscopy and Flow Cytometry.

Detailed methods of cell surface staining, intracellular staining with antibody and flow cytometry can be found in *SI Appendix*.

### BLI Assay.

BLI was performed using Octet RED96e (Sartorius, Bohemia, NY) using Ni-NTA biosensors (#18-5101), which is detailed in *SI Appendix*.

### Western Blotting and Southern Blotting.

Western blot signals were visualized with an Odyssey imaging system (LI-COR, Lincoln, NE). Details are provided in *SI Appendix*.

### rAAV Transduction in Mice and in vivo and ex vivo Bioluminescence Imaging.

CD164-KO (CD164^−/−^; C57BL/6JGpt-Cd164^em1Cd10023^/Gpt) mice on C57BL/6JGpt background (strain ID: T016996) and WT mice (CD164^+/+^) were purchased from GemPharmatech USA (SanDiego, CA). Mice at 7 to 8 wk of age were injected via the tail vein with rAAV vectors. At the days postinjection, as indicted in Fig. 7 and *SI Appendix*, Figs. S10 and S11, the mice were anesthetized and in vivo imaged under an IVIS Spectrum system (Xenogen, Revvity). The lungs of rAAV4-injected mice were harvested for ex vivo imaging. Detailed animal studies are provided in *SI Appendix*.

## Ethics Statement

Primary HSAE cells were obtained from the Tissue and Cell Core, University of Iowa, and were deidentified. Institutional Review Board (IRB) reviews were waived.

All animal experiments were conducted in accordance with the guidelines from the University of Kansas Medical Center Institutional Animal Care and Use Committee (IACUC). Prior to study initiation, the protocol was approved by the University of Kansas Medical Center IACUC (Protocol # 24-12-423). Mice were housed in KUMC LAR under pathogen-free conditions in accordance with institutional biosafety and animal welfare standards. Animals were housed in a specific pathogen free room with irradiated food, UV-irradiated and acidified water, and provided with regular bedding changes. All personnel received training in humane handling techniques and proper reporting procedures.

### Statistical Analysis.

All data are presented as mean ± SD obtained from three independent experiments by using GraphPad Prism 10. Statistical significance (*P* value) was determined by using an unpaired Student’s *t* test for two groups or one-way ANOVA with the post hoc Bonferroni test for the comparison among more than two groups. ****P < 0.0001, ***P < 0.001, **P < 0.01, and *P < 0.05 were considered statistically significant, and “ns” represents no statistical significance.

## Supplementary Material

Appendix 01 (PDF)

Dataset S01 (XLSX)

## Data Availability

NGS data are available from the National Center for Biotechnology Information Sequencing Read Archive (SRA) under accession numbers SAMN50433219 (Sort 0), SAMN50433221 (Sort 2), and BioProject under accession number PRJNA1301022 (70). All other data are included in the manuscript and/or supporting information.
