# Supplementary material for: Identification of CD164 as an essential entry receptor for divergent adeno-associated viruses
Source: Proc Natl Acad Sci U S A. 2026 Mar 5;123(10):e2525865123. doi: 10.1073/pnas.2525865123 (PMC12974471; doi:10.1073/pnas.2525865123)
Supplement: Supplementary file 1 — Appendix 01 (PDF) [file pnas.2525865123.sapp.pdf]

Supplementary Information (SI) Appendix

Identification of CD164 as an Essential Entry Receptor for Divergent Adeno-Associated Viruses

Xiujuan Zhang, Donovan Richart, Shane McFarlin, Fang Cheng, Soo Yeun Park, Anwen Zhang-Chen, Richenda McFarlane, Chuan Xiao, Ziying Yan, and Jianming Qiu*

*Corresponding author. Email: jqiu@kumc.edu

This PDF file includes:

SI Materials and Methods

Fig. S1 to S12 and Legends

SI References

Other Supplementary Information for this manuscript includes the following:

Dataset S1

SI Materials and Methods

Cells and cell culture.

HEK293 cells: HEK293 cells (#CRL-1573, ATCC) and HEK293$^{AAVR\text{-}KO}$ cells (1) were grown in Dulbecco's Modified Eagle Medium (DMEM; #SH30022.01, Cytiva, Marlborough, MA) supplemented with 10% fetal bovine serum (FBS) and 100 units/mL penicillin-streptomycin in a humidified incubator with 5% CO_2 at 37°C. FreeStyle 293-F cells (#R79007293F, ThermoFisher, Waltham, MA) were grown in FreeStyle 293-F Expression Medium (#12338026, ThermoFisher). Cells were cultured in shaking flasks on an orbital shaker at 130 rpm in a humidified incubator with 8% CO_2 at 37°C. The cells were maintained at a low density of 0.2-2 million/mL.

Other cell lines: NIH3T3 (#CRL-1658, ATCC), COS7 (#CRL-1651, ATCC), HeLa (#CCL-2, ATCC), and Huh7 cells (CVCL_0336), were grown in DMEM supplemented with 10% FBS and 100 units/mL penicillin-streptomycin in a humidified incubator with 5% CO_2 at 37°C.

Large-airway epithelial cells: CuFi-8 cells are human primary tracheal (large) airway epithelial cells derived from a cystic fibrosis patient and immortalized by the expression of human telomerase reverse transcriptase and human papillomavirus E6/E7 oncogenes (2). They were cultured on collagen-coated T75 flasks in PneumaCult-Ex Plus medium (#05040; StemCell Technologies, Vancouver, BC).

Primary human small-airway epithelial cells: Human primary small-airway epithelial cells, isolated from a healthy donor, were obtained from the Tissue and Cell Core, University of Iowa, and were cultured on collagen-coated T75 flasks in PneumaCult-Ex Plus medium.

Human large- and small-airway epithelium (HAE and HSAE) cultured at an air-liquid interface (ALI): Proliferating CuFi-8 cells or HSAE cells dissociated from flasks and loaded onto Transwell permeable supports (#3470; Costar, Corning, NY) at a density of 1.5 × 10^5 cells per insert in PneumaCult-Ex Plus medium. At 2 to 3 days after seeding, the media were replaced with PneumaCult-ALI medium (#05001; StemCell). The cells were then differentiated/polarized in the ALI medium at an ALI for 3–4 weeks (3). The maturation of the polarized cultures was determined by measuring transepithelial electrical resistance (TEER) with a Millicell ERS 3.0 Digital Voltohmmeter (MilliporeSigma, St Louis, MO). ALI cultures with a TEER value of >1,500 $\Omega \cdot cm^2$ were used in experiments.

Plasmids.

rAAV production plasmids: pAAVRep2-iCap2.4 was constructed by replacement of the PHP.eB VP1u and VP2/3 gene with AAV2 VP1u and AAV4 VP2/3 gene (GenBank accession NC_001829), respectively, in pUCmini-iCAP-PHP.eB (#103005, Addgene, Watertown, MA). pAAVRep2-iCap4, pAAVRep2-iCap5, pAAVRep2-iCapBAAV, and pAAVRep2-iCapCslAAV were constructed by replacing the PHP.eB VP1 gene with the VP1 gene of AAV4, AAV5 (4), BAAV (#NC_005889) and CslAAV (#JN420371), respectively, in pUCmini-iCAP-PHP.eB. rAAV transgene plasmid pAVF5tg83fLuc-CMVmCherry(4.6) and the adenovirus (Ad) gene helper plasmid pHelper have been described previously (5). In some vectors, transgene plasmids, pscAVtg83nLuc-Ef1aGFP and pAAVdual-CMVeGFP-T2A-fLuc were used. The former was constructed by replacing the U6-gRNA cassette with tg83

promoter-driven nano luciferase (nLuc) gene in pTRdgPuroEf1a.eGFP (6). The latter was constructed by inserting a T2A-fused firefly luciferase (fLuc) gene downstream of the GFP gene in pAAVdual-CMV-eGFP (#230931, Addgene) that also contains the Ad miniHelper element (7).

Lentiviral vector: Guide(gRNA)-expressing lentiviral vectors for gene KO were constructed by inserting the targeting sequences of single guide (sg)RNAs into lentiCRISPRv2 puro (#52961) or lentiCRISPRv2 neo (#98292, Addgene). The gRNA sequence targeting to *CD164* is 5'- CAG TTA GTG ATT GTC AAG TG-3', which was inserted into lentiCRISPRv2. To target *MRE11*, three gRNAs: 5'-AAT GCT GAA CGG GAA CGT CT-3', 5'-GCA ATC ATG ACG ATC CCA CA-3', and 5'-GGC AAT CAT GAC GAT CCC AC-3' were inserted into lentiCRISPRv2 neo, respectively. A non-targeting (NT) gRNA-expressing vector has been described previously (8).

CD164 expression plasmids: CD164 ORF (#AF263279) and the mutants, ∆MD1, ∆MD2, ∆CRD, ∆MD1+2, N104Q, and SP(CD34) were codon-optimized with a HA tag at the C-terminus, and synthesized at Twist Biosciences (South San Francisco, CA). Mouse CD164 (#AF299345) was synthesized at Twist Biosciences (South San Francisco, CA). They were cloned into pLentiCMV-Blast-empty (#17486, Addgene).

Lentivirus production and transduction.

Lentiviruses were produced by transfecting HEK293T cells with lentiviral vectors, along with two packaging plasmids, psPAX2 and pMD2.G, using PEI MAX, concentrated, and titrated as described previously (8,9). Cells were transduced at a multiplicity of infection (MOI) of 5 transduction units per cell as described previously (8).

rAAV production.

rAAV vectors were produced by transfection in Viral Production Cells (VPC) 2.0 (ThermoFisher) with triple plasmids (Rep2-iCap helper, transgene plasmid, and Ad pHelper) or with dual plasmids (Rep2-iCap helper and the pAAVdual) at equal molar ratios, using AAV-MAX Helper-Free AAV Production System Kit (ThermoFisher). Vectors were purified using two rounds of CsCl gradient ultracentrifugation followed by dialysis against phosphate buffered saline (PBS, pH7.4) (5,10). The purified vectors were quantified by quantitative (q)PCR using a transgene-specific probe as DNase-resistant particles (DRP), as previously described (5). rAAV1, 2, 3B, 6, 7, 8, 9, 11, 12, and 13, and AAVrh74 vectors that package a CMV-driven fLuc gene were purchased from AAVnerGene (Rockville, MD). rAAVrh32.33(CMVeGFP-T2A-fLuc) (#INV41) was purchased from Gene Transfer Vector Core, Mass General Brigham.

CRISPR/Cas9-based gene KO.

HEK293, Huh7, 293-F, primary HSAE and CuFi-8 cells were transduced with lentiviral vectors expressing specific gene targeting gRNAs. Transduced cells were selected with antibiotics (puromycin or neomycin based on the vector used). KO efficiency was confirmed by protein expression or genomic sequencing. The gene KO HSAE or CuFi-8 cells were then differentiated at an ALI to generate polarized airway epithelial ALI cultures.

gRNA library and genome-wide CRISPR/Cas9 screen.

A genome-wide CRISPR/Cas9 screen was conducted in MRE11-KO 293-F cells. Cells were transduced with a lentiviral vector carrying spCas9 and a blasticidin resistance gene (lentiCas9-Blast; #52962-LV, Addgene) and selected with blasticidin (10 µg/mL). Cas9-expressing cells (1×10^8) were then transduced with the Brunello lentiCRISPR gRNA library (#73178-LV, Addgene) and selected with puromycin (2 µg/mL). The double-resistant Cas9/sgRNA-expressing $293F^{MRE11\text{-}KO}$ cells were expanded for rAAV2.4 transduction at an MOI of 50K DRP/cell, followed by FACS using FACSAria III (BD Biosciences, San Jose, CA) to isolate the GFP-negative cells. After two rounds of selection, the genomic DNA (gDNA) from the sorted cells was extracted for next-generation sequencing (NGS).

gDNA extraction, NGS, and bioinformatics analysis.

The cells of the unsorted control ($gDNA^{Sort0}$) and the second ($gDNA^{Sort2}$) sorted groups were subjected to extraction of gDNA using the Blood and Cell Culture DNA Midi Kit (#13343; QIAGEN, Germantown, MD). The gDNA samples were subjected to PCR-based amplification of guide sequences and indexed according to the protocol from the Broad Institute of MIT and Harvard (11). The PCR amplicons were sequenced on an Illumina NextSeq 2000 platform. NGS data were analyzed using the MAGeCK software package for sgRNA recognition sequences (12). Significance values were determined after normalization to the control population, and the data were reported as $-\log_{10}$ (Enrichment score). The data analyzed were visualized using Prism 10 (GraphPad). Genes were categorized by gene ontology (GO) terms using PANTHER v19.0 (13,14). The hits, represented by the enrichment score, were plotted along the y-axis and arbitrarily scattered within their categories along the x-axis. The size of the dot was determined based on the significance between sorted and unsorted groups.

rAAV Transduction *in vitro*.

For monolayer cultured cells, the cells were seeded overnight in 48-well plates. rAAV was added to each well at an MOI as described in each figure legend. At 3 days post-transduction (dpt), the expression/activity of fLuc or nLuc was quantified using the Luciferase Assay System or Nano-Glo Luciferase Assay System (E1483 or #N1110, Promega, Madison, WI) on a Synergy LX Reader (BioTek, Santa Clara, CA).

For the transduction of ALI cultures, 100 µL of rAAV4 diluted in Dulbecco's Phosphate Buffered Saline (D-PBS, pH7.4; #SH30028.03, Cytiva) was added to the apical chamber of the transwell at an MOI of 100K DRP/cell. Subsequently, 0.5 mL of culture media was added to the basolateral chamber. In some cultures, Dox was added at a final concentration of 2 µM in the basolateral media. After ~16 h, the inoculum in the apical chamber and media in basolateral chambers were removed, and the transwell inserts were washed three times with D-PBS. Fresh culture media were then added to the basolateral chamber.

rAAV vector binding and entry assays.

We carried out the vector binding and entry assays as previously published (8). Briefly, for binding assays, cells were incubated with rAAV vectors at 4°C for 2 h. Unbound virions were removed by washing with D-PBS, and cells were lysed for qPCR quantification of bound

vector genomes. In entry assays, cells were incubated with rAAV at 37°C for 2 hours, washed, and treated with neuraminidase (#11585886001, MilliporeSigma) at 50 mU/mL for 1 h to remove cell surface-bound virions. Cells were then detached with trypsin, lysed, and the internalized vector genomes were quantified by qPCR. Total DNA was extracted from the cells using the DNeasy Blood & Tissue Kit (Qiagen, Hilden, Germany). Vector genomes were quantified by qPCR using primers specific to the rAAV genome (transgene: e*GFP*) as described previously (5).

Immunofluorescence assay and confocal microscopy.

Cell surface staining with lectin: Cells were fixed with 4% paraformaldehyde and blocked with carbo-free blocking solution (#SP-5040-125, Vector Laboratories, Newark, CA). The cells were then incubated with biotinylated *Maackia amurensis* lectin II (MAL II) (#B-1265, Vector Laboratories) for 30 min at 4°C. After washing, cells were stained with fluorophore (FITC) -conjugated streptavidin (#SA-5001-1, Vector Laboratories), permeabilized, and co-stained with primary/secondary antibodies as indicated.

Intracellular staining with antibody: Cells were fixed with 4% paraformaldehyde, permeabilized with 0.1% Triton X-100, and blocked with 5% bovine serum albumin (BSA) in PBS. Cells were then incubated with primary antibodies followed by fluorescently conjugated secondary antibodies. Nuclei were stained with DAPI. Images were captured using a confocal microscope (CSU-W1 SoRa, Nikon, Melville, NY).

Flow cytometry.

Cell surface staining with antibody: ~2 million cells were washed twice with D-PBS and dissociated using Accutase (#A6964, MilliporeSigma). Single-cell suspensions were incubated with a mouse monoclonal anti-CD164 antibody (clone 67D2) for 20 min on ice. After primary antibody incubation, cells were washed three times with ice-cold D-PBS and then incubated with an Alexa 488-conjugated anti-mouse secondary antibody (ThermoFisher) for 20 min on ice.

Cell surface staining with lectin: ~2 million cells were washed twice with D-PBS, dissociated using Accutase, and blocked with carbo-free blocking solution (#SP-5040-125, Vector Laboratories, Newark, CA). The cells were then incubated with fluorescein isothiocyanate (FITC)-conjugated Wheat Germ Agglutinin (WGA) (#FL-1021) or FITC-conjugated Jacalin (#FL-1151-5, Vector Laboratories) for 15 min on ice.

Intracellular staining with antibody: ~2 million cells were fixed, permeabilized, and incubated with either an anti-AAV4 (intact particle) mouse monoclonal antibody (#610147, clone ADK4, ARP, Waltham, MA, USA) or an anti-CD164 primary antibody (clone 67D2) for 30 min at room temperature. After washing cells were stained with Alexa Fluor 647-conjugated goat anti-mouse IgG (H+L) cross-adsorbed secondary antibody (# A-21235) or an Alexa 488-conjugated anti-mouse secondary antibody (#A-11001, ThermoFisher) for 20 min.

Following three additional washes with ice-cold DPBS, stained cells were analyzed using a 5-laser spectral flow cytometer (Aurora; Cytek Biosciences, Seattle, WA). Data were processed and analyzed using FlowJo v10 software (FlowJo LLC, Ashland, OR).

Biolayer interferometry (BLI) assay.

BLI was performed using Octet RED96e (Sartorius, Bohemia, NY). Ni-NTA biosensors (#18-5101) were pre-equilibrated in assay buffer (25 mM Tris-HCl, 150 mM NaCl, pH7.5) for 10 min before immobilizing with a polyhistidine (His)-tagged protein at 35 µg/mL in kinetic buffer for 300 seconds (s) to allow proper binding. A baseline reading was recorded after protein loading in assay buffer for 60 s to ensure stability. The biosensors were dipped into wells containing different rAAV serotypes at varying concentrations for 300 s. The binding of rAAV vectors to the immobilized protein was monitored in real-time. The biosensors were transferred back into the assay buffer, and the dissociation of rAAV vectors from the protein was recorded for 600 s.

To assess the effect of pH on CD164 binding to rAAV, association and dissociation phases were performed in the assay buffers pre-adjusted to pH 7.4, 7.0, 6.5, and 5.5, respectively. Raw BLI sensor data were analyzed using Octet Data Analysis Software for kinetic parameters: association rate constant (ka), dissociation rate constant (kd), and equilibrium dissociation constant (K_D).

Western blotting.

Cells were collected and lysed as previously described (15,16). The lysates were separated by sodium dodecyl-sulfate polyacrylamide gel electrophoresis (SDS-PAGE) and transferred to a polyvinylidene difluoride membrane. The membrane was blocked with 5% non-fat milk in Tris-buffered saline buffer, pH7.4, and incubated with a primary antibody, followed by infrared dye-conjugated IgG secondary antibody. Finally, the membrane was imaged on a LI-COR Odyssey F imager (LI-COR Biosciences, Lincoln, NB).

rAAV transduction in mice.

CD164 KO (CD164$^{-/-}$; C57BL/6JGpt-Cd164^{em1Cd10023}/Gpt) mice on C57BL/6JGpt background (strain ID: T016996) and wild-type mice (CD164$^{+/+}$) were purchased from GemPharmatech USA (SanDiego, CA). Mice were genotyped to confirm their genetic status (**Fig. S9**). Mice at 7-8 weeks of age were used to assess the effect of *CD164* KO on AAV transduction. Mice from each group (CD164$^{+/+}$: n = 4 for each rAAV; CD164$^{-/-}$: n = 4 for each AAV) were injected via the tail vein with 1 × 10^{11} DRP of vector that carries rAAV-CMVGFP-T2A-fLuc in 100 µl of D-PBS.

***In vivo* and *ex vivo* bioluminescence imaging.**

To visualize rAAV transduction, at the indicated days post-injection, the mice were anesthetized with 2% isoflurane in oxygen, and D-luciferin substrate (#122799, Revvity, Waltham, MA) was injected intraperitoneally at a dose of 150 mg/kg. Three untransduced mice were also injected with D-luciferin and used as background controls. After a 10-min incubation period, mice were placed in a light-tight chamber, and *in vivo* bioluminescence images were captured using an IVIS Spectrum system (Xenogen, Revvity), equipped with a cryogenically cooled charge-coupled device camera. For rAAV4-injected mice, at 15 days post-injection, the mice were anesthetized and injected with D-luciferin substrate. After ~10 min, mice were euthanized, and the lung was harvested for *ex vivo* bioluminescence imaging. The images were generated as false-color representations, where the highest bioluminescence is shown in

red and the lowest in dark blue. The average radiance (photons emitted per second per cm^2) was quantified.

For each time point, a consistent 'region of interest' (ROI) was defined surrounding each mouse/lung to quantify the radiance (photons/ sec/cm^{-2} /sr) produced by luciferase activity. The mean and standard deviation (SD) of the radiance measurements were calculated for each experimental group at each time point.

Purified proteins used in this study.

$rCD164^{ECD}$: The extracellular domain (Met 1-Asp 162) of human CD164 was fused with a His tag at the C-terminus, expressed in and purified from HEK293 cells. The purity is ≥95%, as determined by SDS-PAGE (#12260-H08H, Sino Biological, Paoli, PA).

$rCD34^{ECD}$: His tagged recombinant human CD34 ECD (Met 1-Thr 290) was expressed with a His tag at the C-terminus and purified from HEK293 cells (#10103-H08H, Sino Biological).

$rCD164^{ECD\text{-}E}$: His-tagged ECD of human CD164 was purified from *E. Coli* (#ab134891, Abcam, Waltham, MA, or custom made at Biomatik, Ontario, Canada).

Antibodies used in this study.

Primary antibodies: Anti-CD164 monoclonal antibodies, Clone N6B6 (#551296) and Clone 67D2 (#324802), were purchased from BD Biosciences (San Jose, CA) and Boilegend, Inc. (East Brunswick, NJ), respectively. A polyclonal anti-CD164 antibody recognizing residues D99–V149 (#PA5104047) and a biotinylated anti-AAVX antibody (#7103522100) were obtained from Thermo Fisher Scientific. Rabbit anti-CD164 (#12083-2-AP) was sourced from Proteintech (Rosemont, IL). Mouse anti-β-actin (#AC004) was obtained from ABclonal (Woburn, MA). Rabbit monoclonal antibodies against Rab5 (C8B1, #3547S), Rab7 (D95F2, #9367S), were obtained from Cell Signaling Technology (Danvers, MA). Anti-HA (#H9658) was obtained from MilliporeSigma. Anti-AAV4 (intact particle) mouse monoclonal antibody (#610147, clone ADK4) was purchased from American Research Products (Waltham, MA). Rabbit polyclonal anti-TGN46 antibody (#NBP1-49643) was obtained from Novus Biologicals (Centennial, CO).

Secondary antibodies: Alexa Fluor 488-conjugated Goat anti-Mouse IgG (H+L) cross-absorbed secondary antibody (# A-11001), Alexa Fluor 647-conjugated goat anti-mouse IgG (H+L) cross-absorbed secondary antibody (# A-21235), and Alexa Fluor 594-conjugated goat anti-rabbit IgG (H+L) cross-adsorbed secondary antibody (#A-32754) were purchased from Thermo Fisher Scientific. DyLight 800-conjugated anti-rabbit IgG (#5151S), DyLight 800-conjugated anti-mouse IgG (#5257S), DyLight 680-conjugated anti-rabbit IgG (#5366S) and DyLight 680-conjugated anti-mouse IgG (#5470S) were purchased from Cell Signaling (Danvers, MA). Fluorescein-conjugated streptavidin (SA-5001) was obtained from Vector Laboratories (Burlingame, CA).

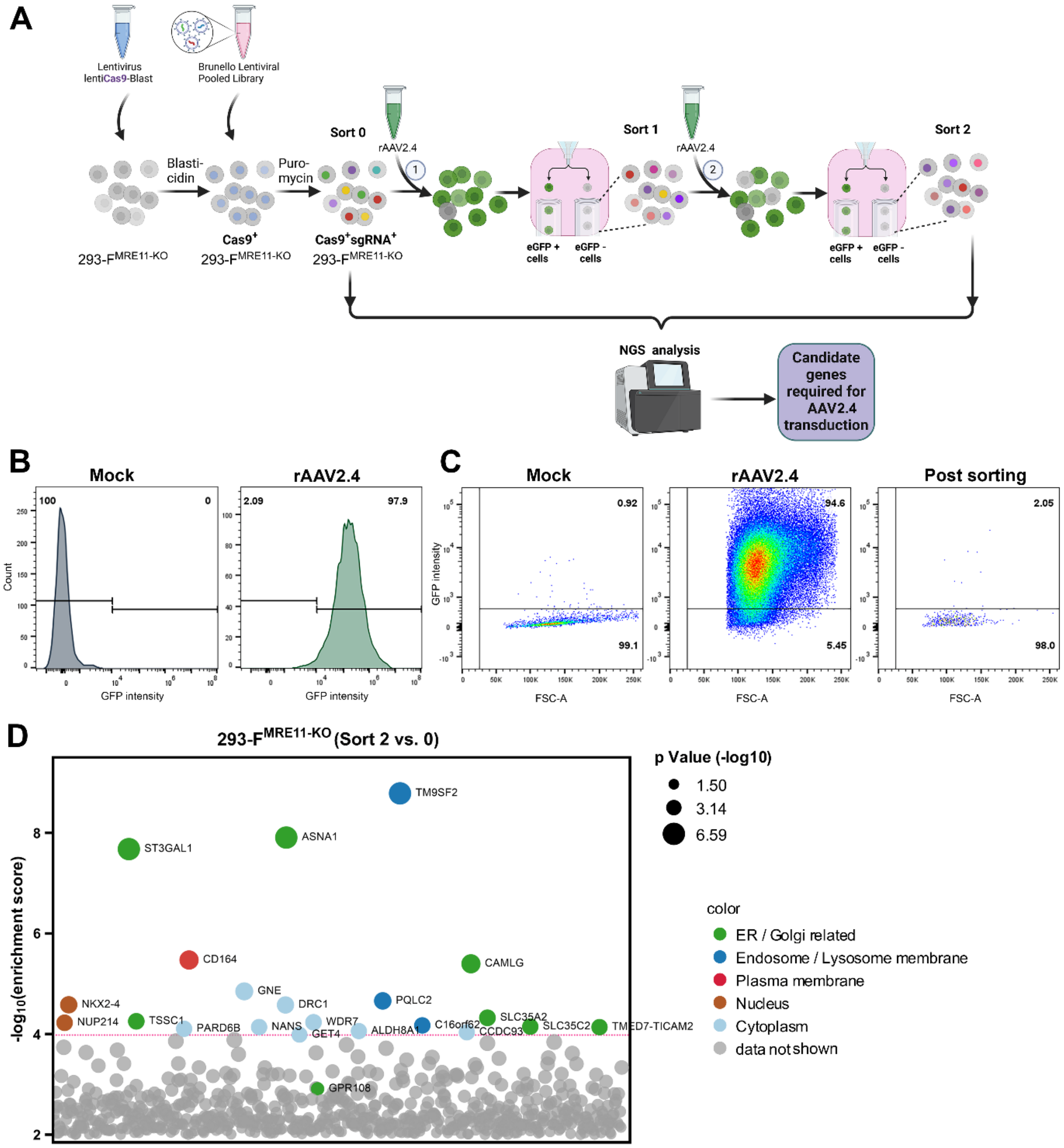

Fig. S1. Genome-wide CRISPR/Cas9 knockout screening identifies host factors that restrict rAAV4 transduction.

(A) A diagram of a genome-wide CRISPR/Cas9 gRNA library screen. Suspension 293-F$^{MRE11\text{-}KO}$ cells were transduced with a spCas9-expressing lentiviral vector, lentiCas9-Blast, followed by blasticidin selection. The spCas9-expressing cells ($1{\times}10^8$) were then transduced with the Brunello lentiCRISPR gRNA lentiviral library and selected with puromycin for Cas9/sgRNA-expressing 293-F$^{MRE11\text{-}KO}$ cells. The obtained double-resistant cells were expanded to 2×10^8, of which half were harvested for genomic DNA (gDNA) extraction as the control (gDNA^{Sort0}), while another half were transduced with GFP-expressing rAAV2.4. At 3 dpt, the transduced cells were subjected to FACS and the bottom fraction of the GFP-negative (GFP$^-$) cells was collected and expanded as the Sort 1 cells.

They underwent a 2nd round of screening to enrich the cells resistant to rAAV2.4 transduction. The GFP⁻ cells from the 2nd sort were expanded to 1×10^8 for gDNA extraction ($gDNA^{Sort2}$). The gDNA samples were subjected to NGS and bioinformatics analysis. **(B) rAAV4 transduction in 293-$F^{MRE11-KO}$ cells.** 293-$F^{MRE11-KO}$ cells were transduced with GFP-expressing rAAV2.4, or mock-treated (Mock). The percentage of transduced cells was assessed by flow cytometry. **(C) Selection of rAAV2.4-untransduced cells.** spCas9-expressing 293-$F^{MRE11-KO}$ cells were transduced with the Brunello lentiCRISPR gRNA lentiviral library, followed by transduction of GFP-expressing rAAV2.4. At 3 dpt, GFP-negative (GFP^-) cells were enriched by two rounds of sorting. The first sort collected the bottom 5% GFP^- cells shown (rAAV2.4). After expansion, the cells were infected with rAAV2.4 and sorted again. The secondary sorted GFP^- cells were re-analyzed by flow cytometry (Post sorting). FSC-A: Forward Scatter Area. **(D) Enrichment of target genes in GFP^- cells.** sgRNAs enriched in the GFP^- cell population through two rounds of screening were identified by NGS. MAGeCK analysis identified significantly enriched genes whose KO inhibits rAAV2.4 transduction from the second sort, visualized by enrichment score (y-axis) and statistical significance (circle size). Genes are color-coded according to their Gene Ontology classification. The x-axis represents genes targeted by the Brunello library. Only genes with enrichment scores >10^4 are shown.

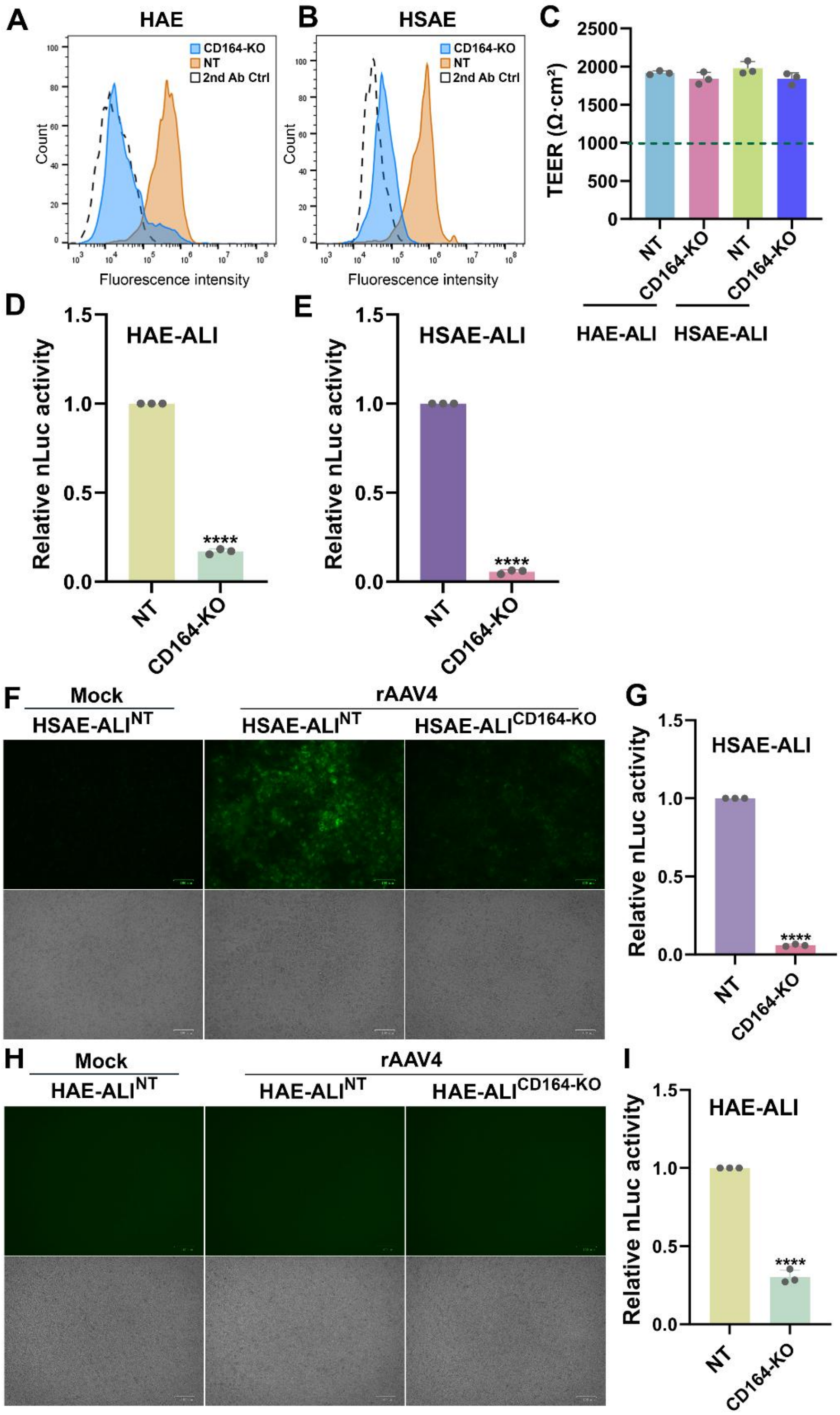

Fig. S2. CD164 is required for efficient transduction of rAAV4 in human airway epithelium.

(A&B) Flow cytometry of CD164 expression on the cell surface. The cells dissociated from HAE-ALI$^{CD164\text{-}KO}$, HSAE-ALI$^{CD164\text{-}KO}$, and their respective NT control cultures were intracellularly stained with α-CD164 and Alexa 488-conjugated secondary antibodies. The histograms show the intensity of the Alexa 488 staining on the x-axis and the number of cells at each intensity level on the y-axis. **(C) Transepithelial electrical resistance (TEER)**

measurement. TEER values of the HAE-ALI and HSAE-ALI cultures, as indicated, were determined in Ω·cm². **(D&E) rAAV4 transduction with doxorubicin (Dox) treatment.** (D) HAE-ALI$^{CD164\text{-}KO}$ and HAE-ALI^{NT} cultures or (E) HSAE-ALI$^{CD164\text{-}KO}$ and HSAE-ALI^{NT} were apically transduced with rAAV4 overnight in the presence of Dox in the basolateral media at a final concentration of 2 μM. At 5 dpt, the cultures were lysed for quantification of nLuc activity. **(F-I) rAAV4 transduction without Dox treatment.** HAE-ALI$^{CD164\text{-}KO}$ and HAE-ALI^{NT}, as well as HSAE-ALI$^{CD164\text{-}KO}$ and HSAE-ALI^{NT} cultures, were infected with rAAV4(tg83nLuc-E1faGFP) at an MOI of 50K DRP/cell. At 5 dpt, the cultures were imaged for GFP expression (F&H) and lysed for nLuc activity (G&I) in HSAE-ALI (F&G) and HAE-ALI (H&I), respectively. *****, P < 0.0001.

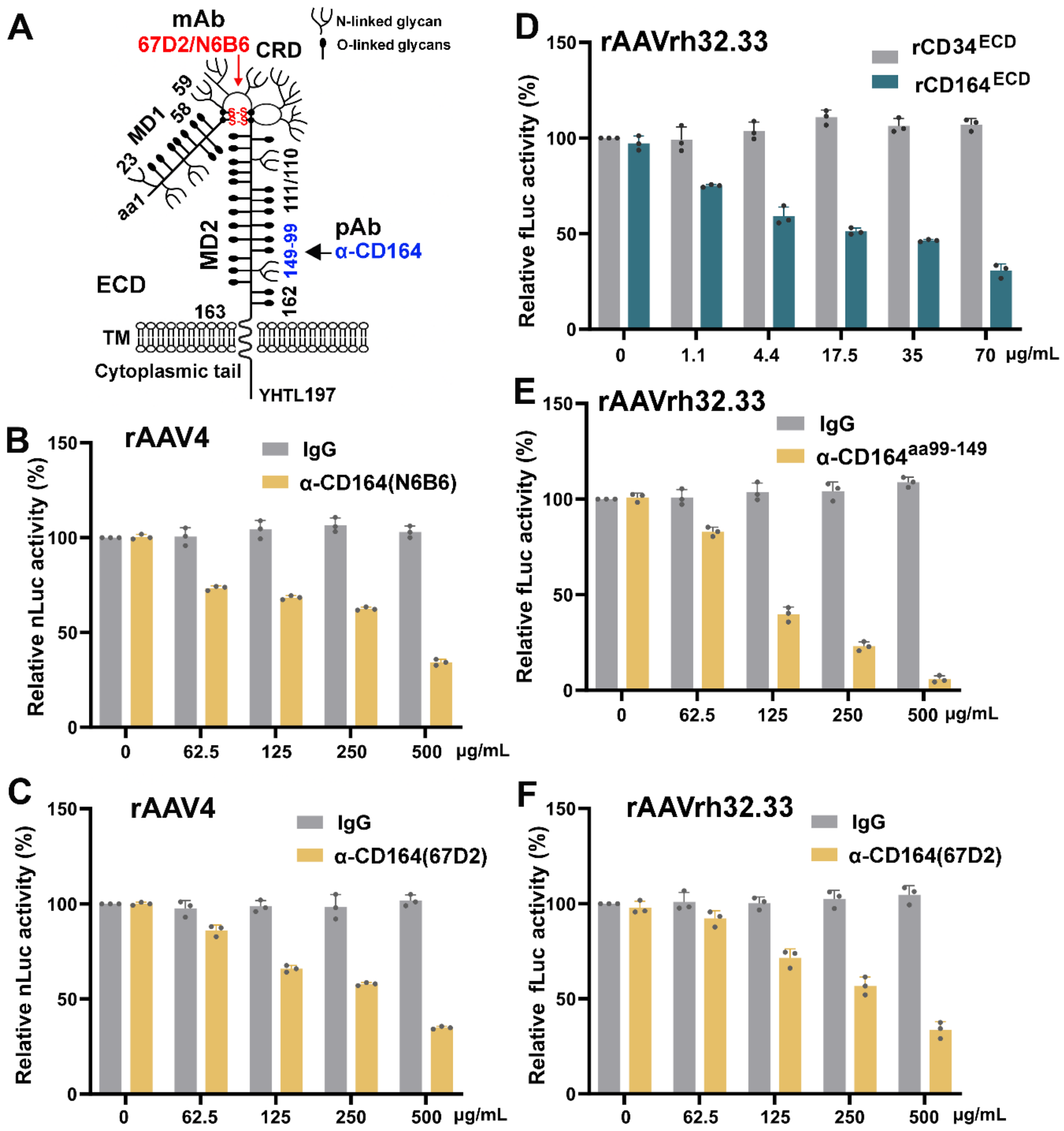

Fig. S3. CD164-targeting monoclonal antibodies inhibit transduction by rAAV4 and rAAVrh32.33 in a dose-dependent manner.

(A) Schematic representation of human CD164 domain structure and the targeting antibodies. A CD164 monomer is diagramed with indicating regions of the signaling peptide, mucin domain (MD1), cysteine-rich domain (CRD), mucin domain (MD2), transmembrane domain (TM), and cytoplasmic tail (CTL), and putative intra-molecular disulfide bridges (S-S). Locations of N-linked (black circles) and O-linked glycans (red circles) are indicated. The epitopes recognized by monoclonal antibodies 67D2 and N6B6 are mapped to the cysteine-rich domain (CRD) region. The polyclonal antibody raised by immunization of a 50 aa epitope (aa99-149 of CD164) is indicated. **(B&C) Inhibition of rAAV4 transduction by α-CD164 mAbs N6B6 and 67D2.** HEK293 cells were pretreated with increasing concentrations (0–500 µg/mL) of IgG control (gray), α-CD164(N6B6) (B) or α-CD164(67D2) (C) (gold) at 4°C for 1.5 h, followed by transduction with nLuc-expressing

rAAV4 at an MOI of 50K. nLuc activity was measured at 3 dpi and normalized to the mock-treated control (set as 100% for relative nLuc activity). **(D) Inhibition of rAAVrh32.33 transduction by soluble recombinant CD164 ectodomain (ECD).** AAVrh32.33 at an MOI of 50K was pre-incubated with various concentrations (0–70 µg/mL) of recombinant CD164 ECD (rCD164^{ECD}) or CD34 ECD (rCD34^{ECD}) in serum-free medium at 37°C for 30 minutes. The vector–protein mixtures were then added to HEK293 cells and incubated for 2 h, followed by the addition of fresh media. At 3 dpt, fLuc activity was measured and normalized to the value in mock-treated cells (set up as 100% for relative fLuc activity). The data shown are mean ± SD from three replicates. **(E&F) Inhibition of rAAVrh32.33 transduction by anti-CD164 antibodies.** HEK293 cells were treated with a polyclonal antibody α-CD164$^{aa99-149}$ (E) or a monoclonal antibody α-CD164(67D2) (F) or with IgG control (gray) at 4°C for 1.5 h, followed by transduction with fLuc-expressing rAAVrh32.33 at an MOI of 50K. fLuc activity was measured and normalized to the untreated control (set as 100%). Data are shown with mean ± SD from three repeats.

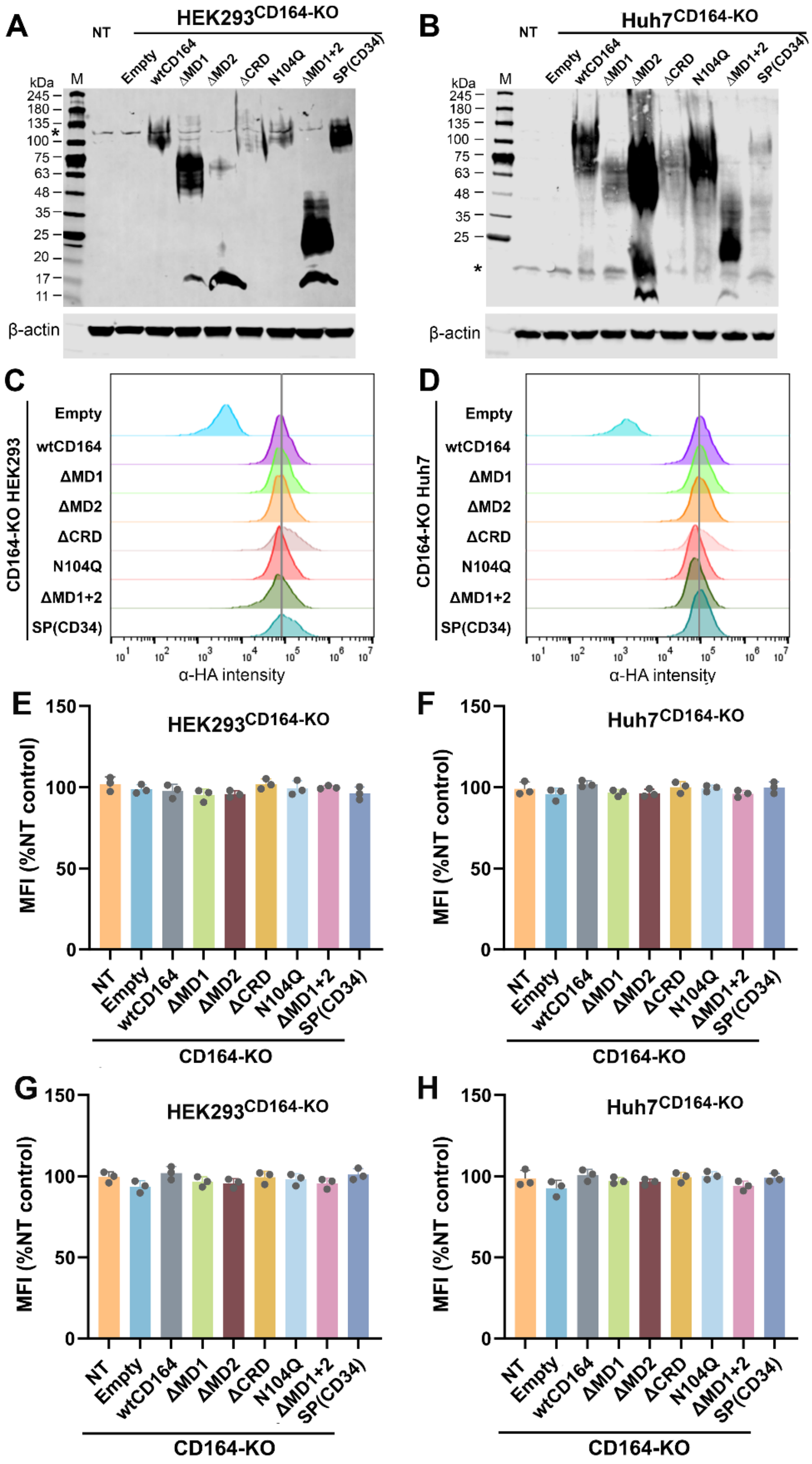

Fig. S4. CD164 ablation and expression of domain mutants do not alter global cell surface glycosylation assessed by lectin staining.

(A-D) Expression of wild-type (wt) and mutant CD164 in CD164 knockout cells. CD164-KO HEK293 cells (A) or Huh7 cells (B) were transduced with lentiviral empty vector (Empty) or the indicated lentiviral constructs expressing wtCD164 or CD164 mutants lacking specific domains, including ΔMD1, ΔMD2, ΔCRD, ΔMD1+2, or the chimeric construct

SP(CD34). After blasticidin selection, the stable expressing cells were collected for the detection of CD164 or mutant expression. (A&B) Western blotting. Cells were analyzed by Western blotting using an anti-HA antibody. β-actin was reprobed as a loading control. The asterisk (*) indicates non-specific bands. Smear bands correspond to various glycosylated forms of HA-tagged CD164. M, protein ladder with sizes in kDa**.** (C&D) Flow cytometry. Cells were fixed and permeabilized, stained with anti-HA antibody, and analyzed by flow cytometry. Overlaid histograms show anti-HA (α-HA) fluorescence intensity (x-axis) for each cell line, with the empty serving as a negative control. Rightward shifts indicate intracellular expression of the corresponding wtCD164 and mutants. **(E-H) Flow cytometry of glycan expression on cell surface.** (E&F) O-glycosylation. FITC-conjugated Jacalin lectin was used to stain the O-glycan on the surface of WT or mutant CD164-expressing HEK293$^{CD164-KO}$ (E) and Huh7$^{CD164-KO}$ (F) cells, followed by flow cytometry. (G&H) Overall glycan expression. N-acetylglucosamine and sialic acid-containing glycan expression on the cell surface was measured using FITC-conjugated wheat germ agglutinin (WGA) staining in HEK293 (G) and Huh7 (H) cells. Mean fluorescence intensity (MFI) was quantified by flow cytometry and normalized to the level in NT control cells. Data represent mean ± SD from three independent experiments.

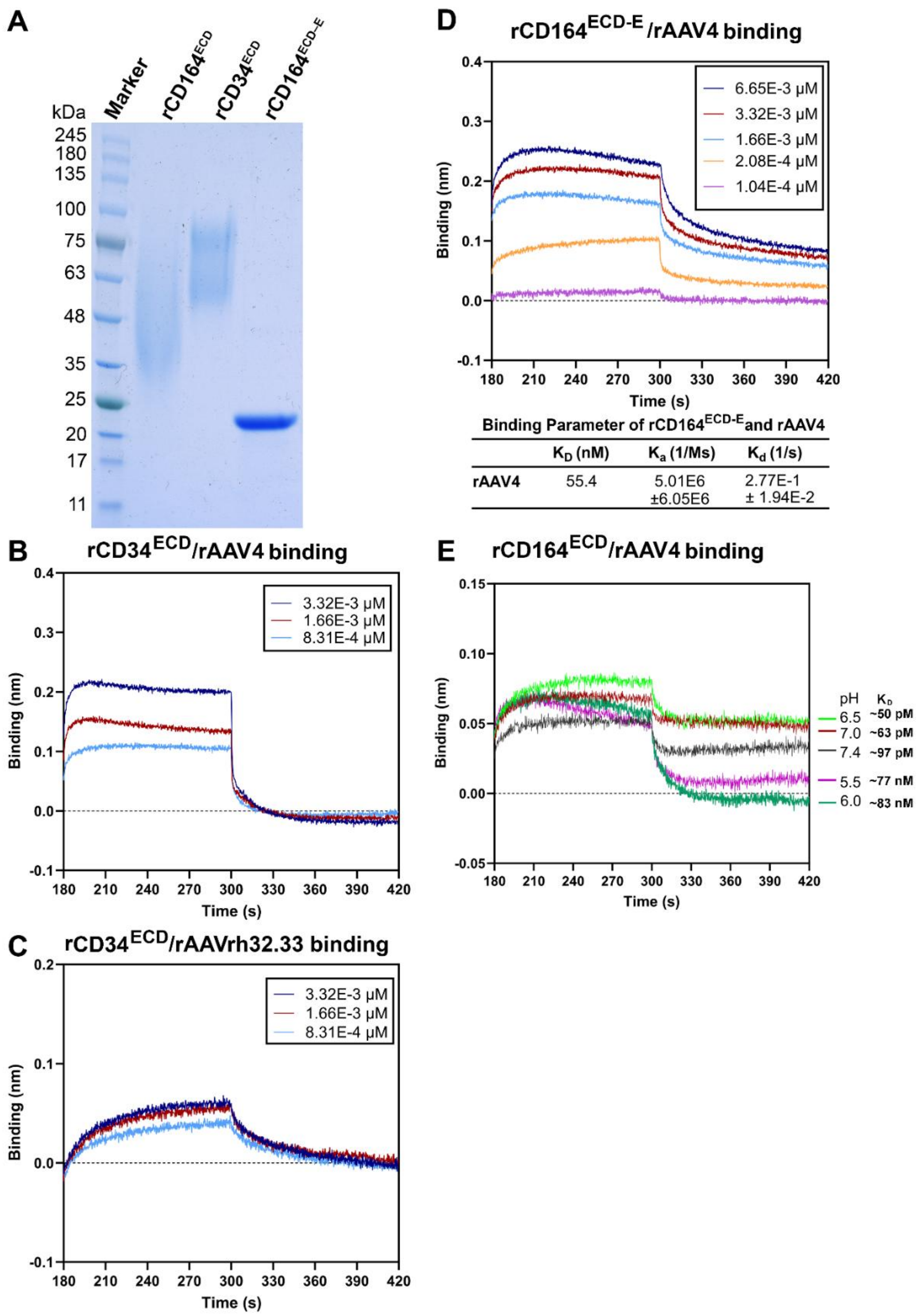

Fig. S5. Biolayer interferometry (BLI) analysis of AAV binding to the extracellular domains of CD34 and CD164.

(A) Analysis of purified recombinant proteins. ~5 μg of each indicated protein was resolved on a 4–20% gradient polyacrylamide gel under reducing conditions and visualized by Coomassie Brilliant Blue staining. Marker: molecular weight marker in kDa; rCD164^{ECD}: recombinant CD164 extracellular domain purified from HEK293 cells; rCD34^{ECD}: recombinant CD34 extracellular domain purified from HEK293 cells. rCD164$^{ECD\text{-}E}$: recombinant CD164 extracellular domain purified from *E. Coli*. **(B&C) Binding of**

recombinant His-tagged CD34 extracellular domain (rCD34^{ECD}) to rAAV. NTA sensors, loaded with rCD34^{ECD}, were used to assess their association and disassociation with rAAV4 (B) and rAAVrh32.33 (C), respectively, at various concentrations as indicated, by BLI. Sensorgrams show dose-dependent binding but not dissociation. **(D) Binding of rAAV4 to rCD164^{ECD-E} expressed in *E. Coli.*** Sensorgrams show binding at increasing AAV concentrations. Equilibrium dissociation constant K_D value represents the ratio of dissociation [k_d (1/s)] and association [k_a (1/Ms)] computed from the real-time binding curves of the protein with rAAV. Kinetic parameters indicate strong binding with nanomolar affinity. **(E) Binding of rAAV4 to rCD164^{ECD} at different pH values**. pH-dependent binding of rAAV4 to CD164 was assessed by BLI. Binding kinetics of rCD164^{ECD} with rAAV4 (at 8.3e-4 μM) were performed in the binding buffer with the indicated pH value. Sensorgrams reveal that binding is pH-dependent, with higher affinity binding observed at pH6.5-7.0 than at pH5.5-6.0.

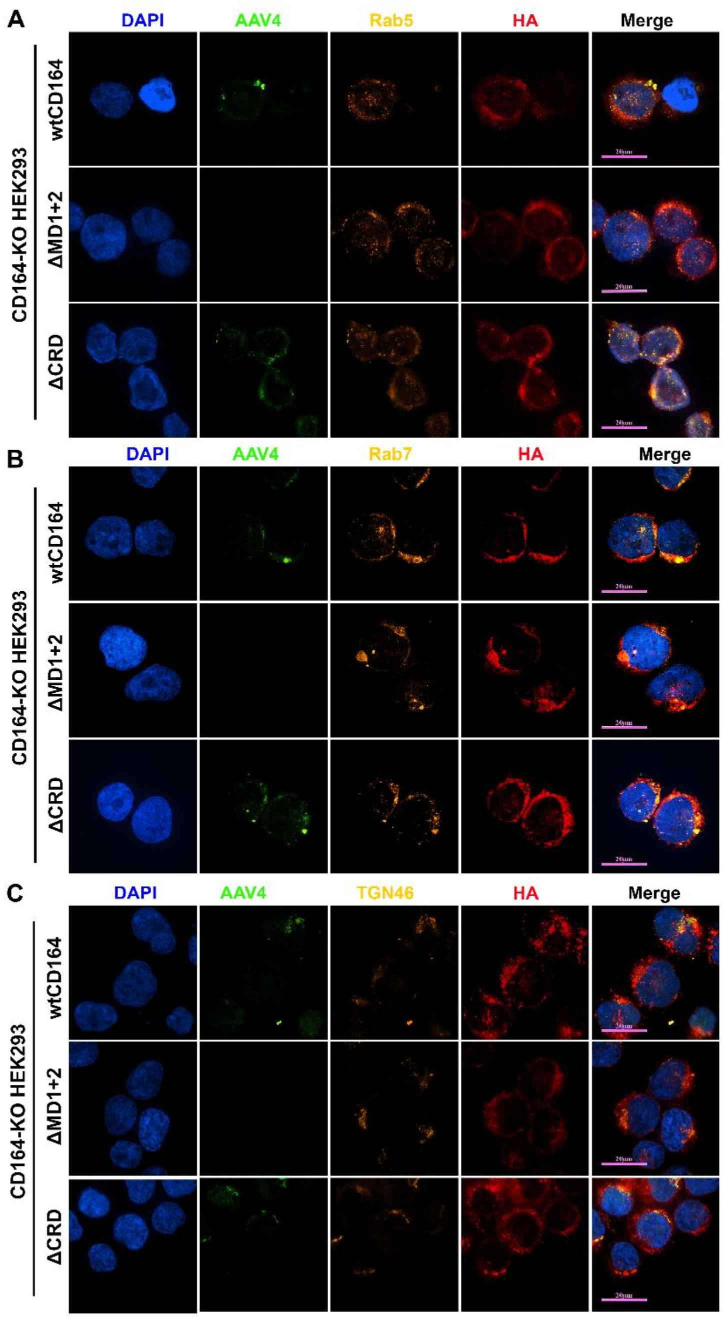

Fig. S6. Colocalization of AAV4 with mutant CD164, endosome and TGN markers.

CD164-KO HEK293 cells were complemented with lentiviral vectors expressing wild-type CD164 (wtCD164), mutant ΔMD1+2, or ΔCRD, followed by blasticidin selection. Cells were then transduced with rAAV4 at an MOI of 50K DRP/cell. **(A) AAV4 co-localizes with Rab5 and CD164.** At 4 hpt, cells are collected and stained to show rAAV4 particles (green), Rab5 (orange) and CD164-HA (red) in the cells. **(B) AAV4 co-localizes with Rab7 and CD164.** At 6 hpt, cells are collected and stained to show rAAV4 particles (green), Rab7 (orange) and CD164-HA (red) in the cells. **(C) AAV4 co-localizes with TGN46 and CD164.** At 8 hpt, cells are collected and stained to show rAAV4 particles (green), TGN46 (orange) and CD164-HA (red) in the cells. Nuclei were stained with DAPI (blue). Images were acquired using a CSU-W1 SoRa spinning disk confocal microscope at 60 × magnification. Bar = 20 μm.

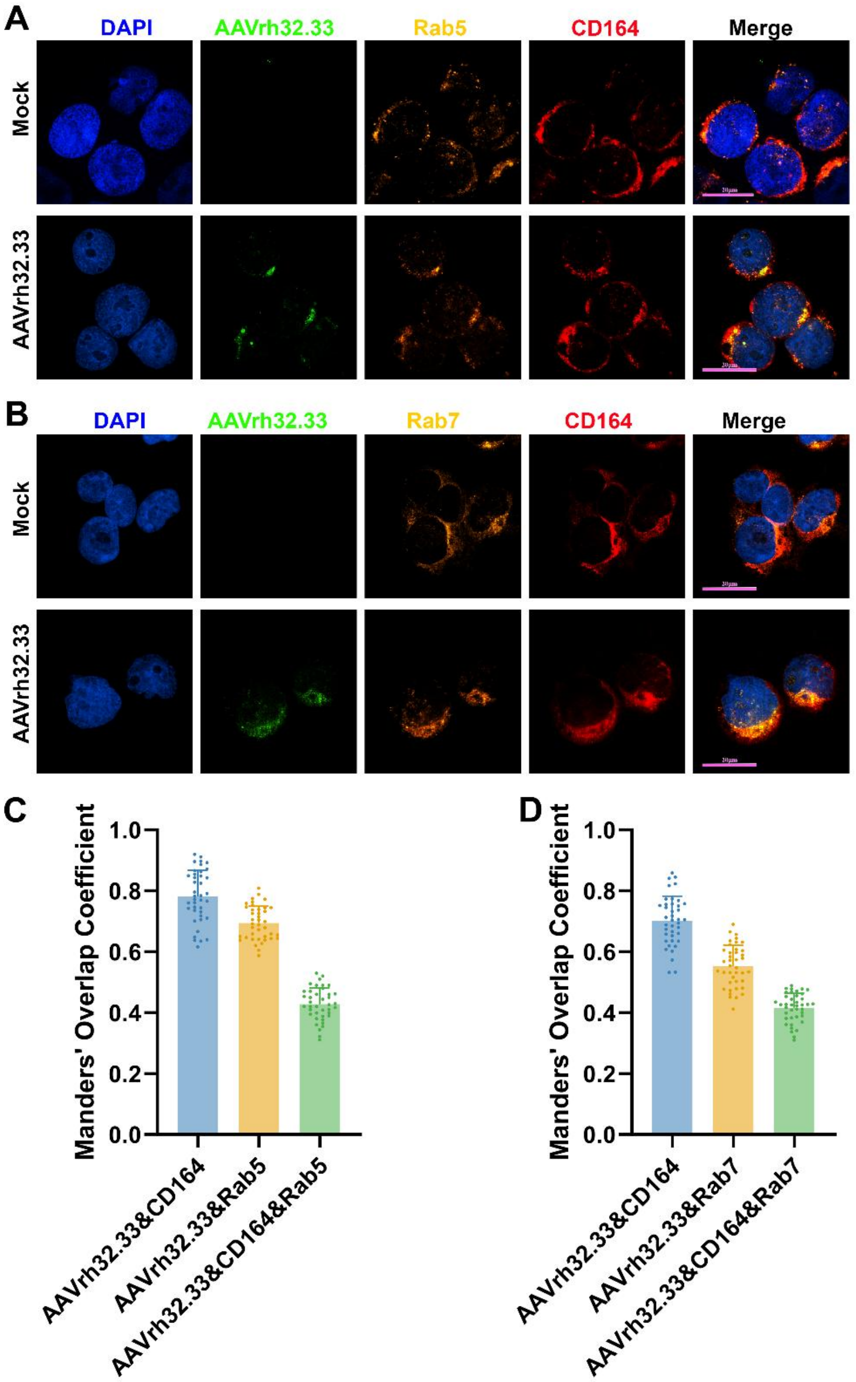

Fig. S7. AAVrh32.33 colocalizes with CD164 during entry and trafficking.

(A&B) Immunofluorescence images. HEK293 cells were transduced with rAAVrh32.33 at an MOI of 50K DRP/cell or mock-infected. At 4 hpt (A) or at 6 hpt (B), the cells were immuno-stained for CD164 (red), AAVrh32.33 capsid (blue), the early or late endosome marker (Rab5 or Rab7; orange) and DAPI (blue). Images were acquired using a CSU-W1 SoRa confocal microscope at 60 × magnification. Bar=20 μm. **(C&D) Quantification of colocalization using overlap coefficient analysis.** Data show the extent of overlap between AAVrh32.33 and CD164, or Rab5/Rab7, or both at 4 hpt (C) or 6 hpt (D). Each dot represents an individual cell; bars represent mean ± SD.

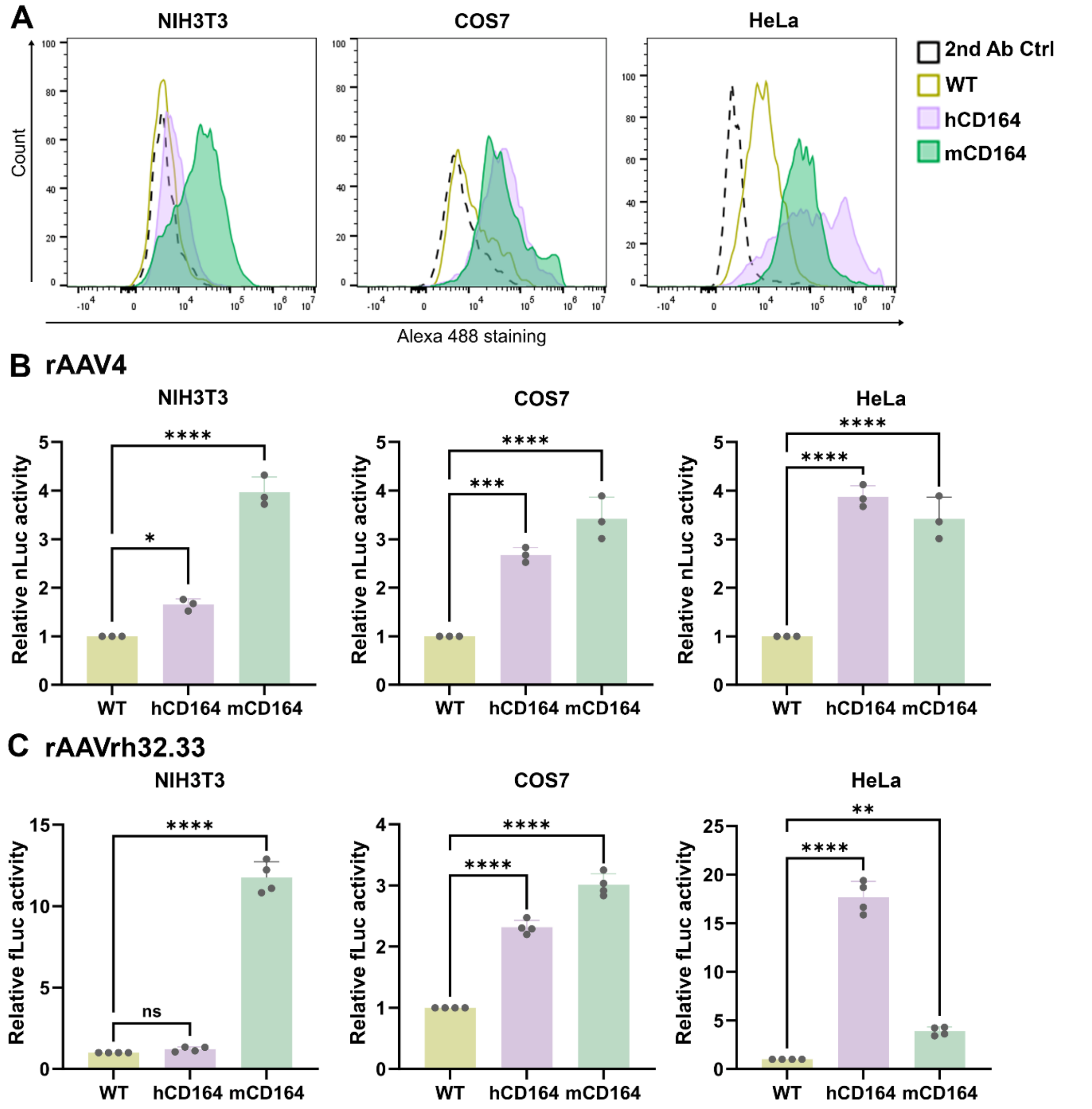

Fig. S8. Overexpression of CD164 boosts Clade G rAAV transduction.

(A) CD164 overexpression. Human CD164 (hCD164) and mouse CD164 (mCD164) were overexpressed in NIH3T3, COS7, and HeLa cells, respectively, by lentiviral transduction, followed by selection with blasticidin. Flow cytometry was used to analyze CD164 expression on the cell surface. **(B&C) rAAV transduction**. Various cell types, as indicated, were transduced with rAAV4 (B) or rAAVrh32.33 (C) at an MOI of 50K. At 3 dpt, luciferase activity was measured and normalized to the value in wild-type (WT; set as 1.0) cells. Data shown are mean ± SD from at least three replicates.

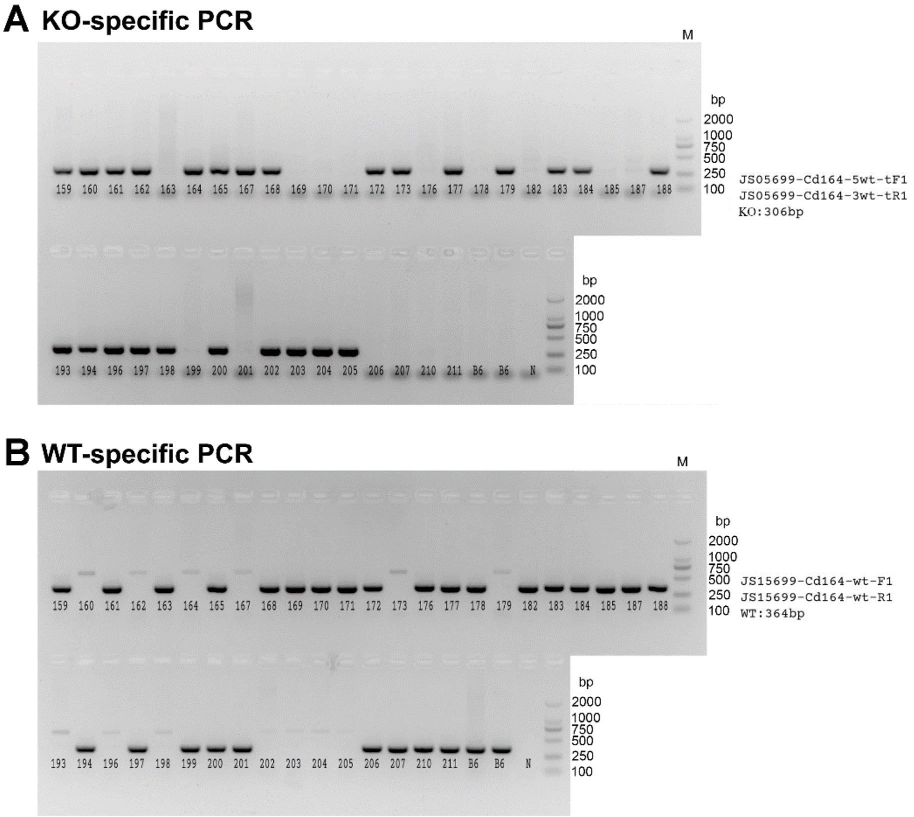

Fig. S9. Genotyping of CD164 knockout mice.

PCR-based genotyping was performed on genomic DNA extracted from tail biopsies of *CD164* KO mice at postnatal day 21. Two separate PCR reactions were used to distinguish wild-type (WT) and knockout (KO) alleles: KO-specific PCR (F1/R1) generated a 306 bp product in KO mice, while WT alleles (~10.2 kb) were not amplified. WT-specific PCR (F2/R2) produced a 364 bp product in WT mice but no product in KO mice. PCR products were separated on a 2% agarose gel and visualized by ethidium bromide staining. Lanes are labeled as follows: P, positive control; WT, wild-type DNA control; B, blank control (ddH$_2$O); M, DNA ladder. Primer sequences: KO primers: F1: 5′-TGT TTG GAG GCA GTG GCT TCTCT -3′; R1: 5′-CAA GGG CTA CCT ACA GCA AGT ACA CAGA -3′. WT primers: F2: 5′-TTC ACT TGA GCT GTT TGG GAA CGTG -3′; R2: 5′-ACC TGG AGT TCA CAG TGA CAC AGA TGA -3′.

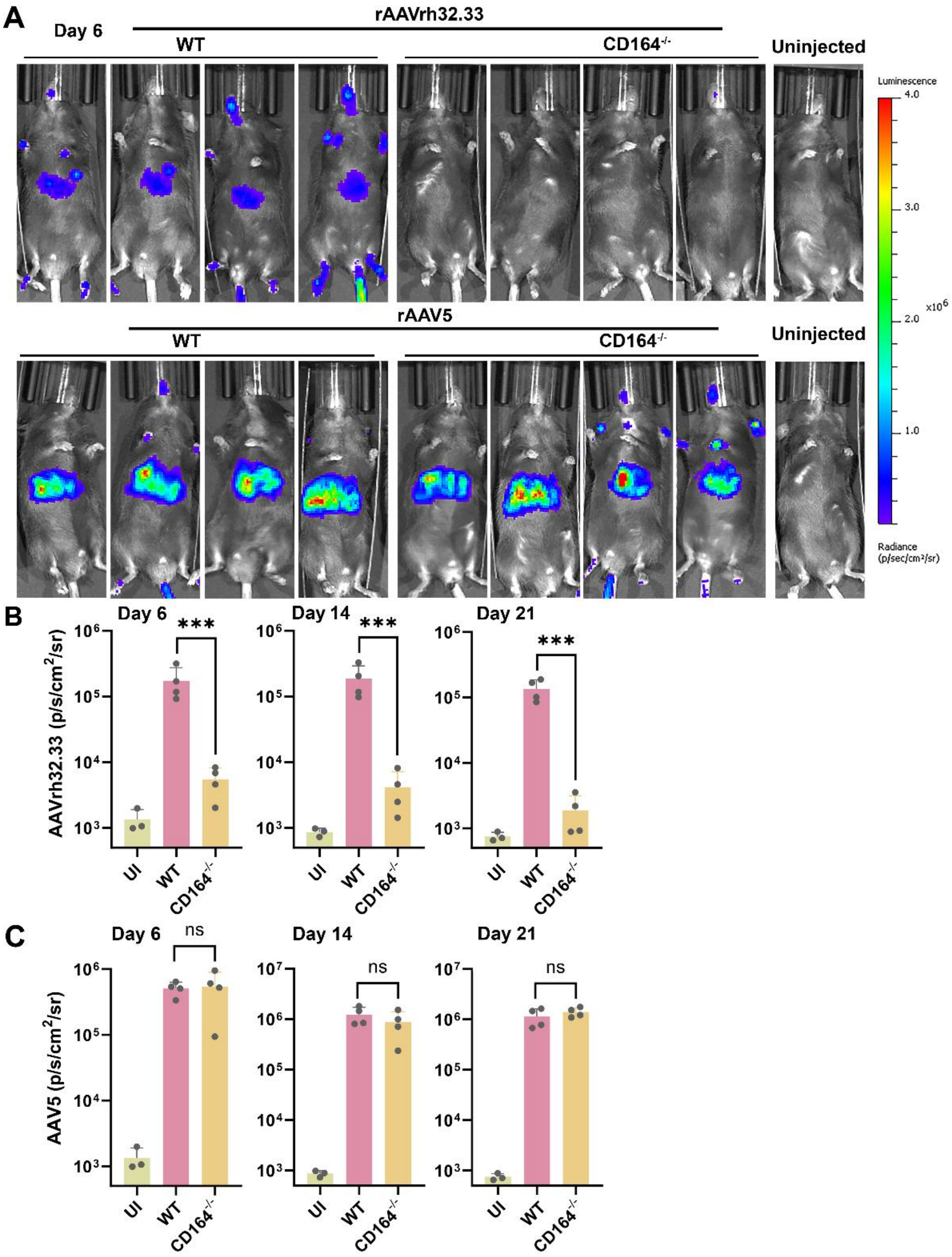

Fig. S10. CD164 is required for efficient *in vivo* transduction by rAAVrh32.33 but not rAAV5.

(A) *In vivo* bioluminescence imaging. WT ($CD164^{+/+}$) and *CD164* KO ($CD164^{-/-}$) C57BL/6JGpt mice were intravenously administrated with fLuc-expressing rAAVrh32.33 (top panel) or rAAV5 (bottom panel) at 1×10^{11} DRP/mouse. At 6 days after injection, mice were imaged under an IVIS Spectrum system following intraperitoneal injection of D-luciferin. One representative animal from the uninjected control group (n=3) is shown on the far right of each panel. Pseudocolored images represent luciferase activity (photons/sec/cm²/sr) with radiance scales shown on the right. **(B&C) Quantification of whole-body bioluminescence signal.** At 6, 14, 21 days post-injection, the imaging signals of the mouse

injected with rAAVrh32.33 (B) and with rAAV5 (C) were quantified. The mean bioluminescence signal of the uninjected (UI) control group represents the background. Each dot represents an individual mouse. Bars show mean ± SD. Statistical significance was determined by one-way ANOVA with multiple comparisons. UI, uninjected; ***, p<0.001; ns, not significant.

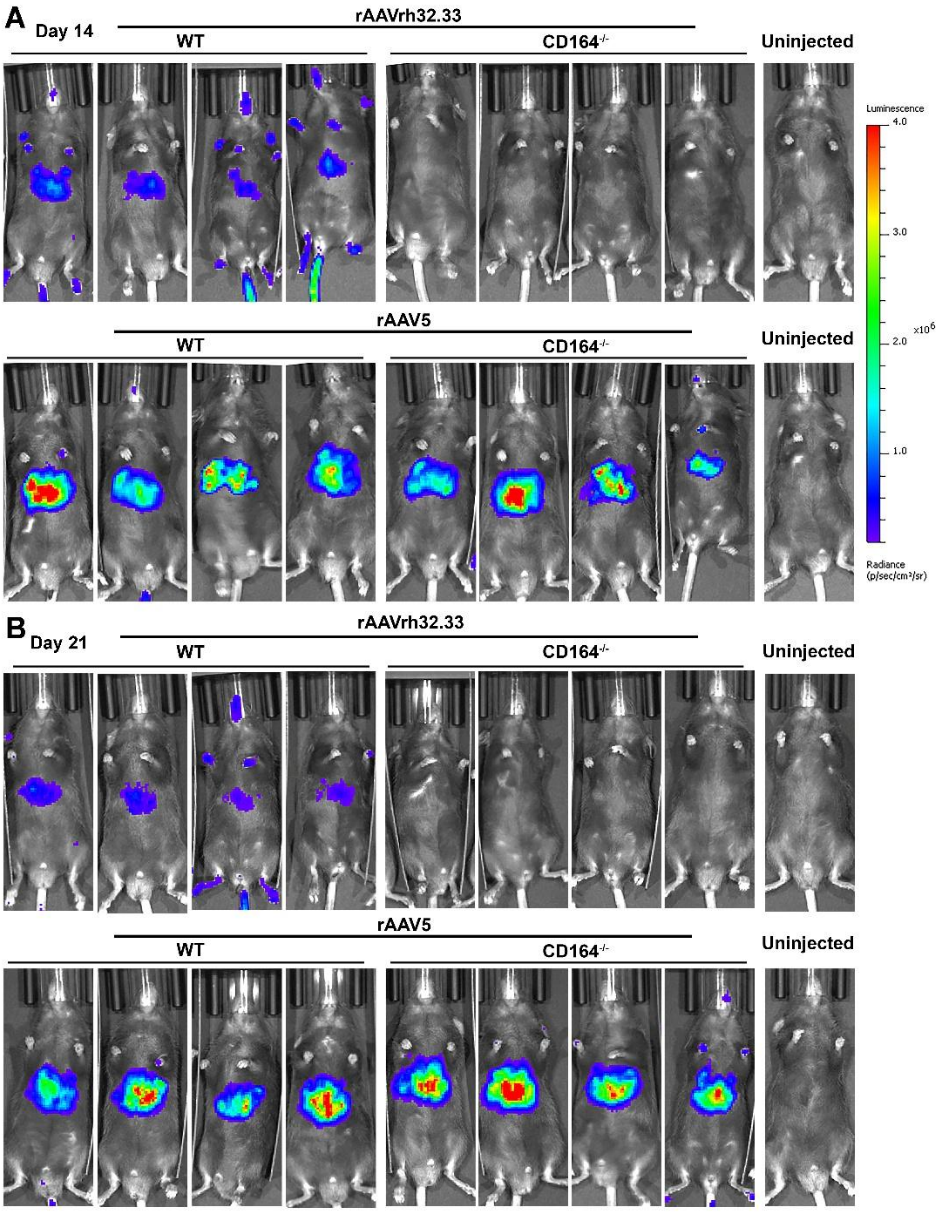

Fig. S11. CD164 is required for efficient *in vivo* transduction by rAAVrh32.33 but not rAAV5 at 2 and 3 weeks post-transduction.

At 14 and 21 days post-AAV injection, the mice in the experiment carried out in **Figure 10** were imaged under an IVIS Spectrum system following intraperitoneal injection of D-luciferin. A representative uninjected control animal is shown on the far right of each panel. Pseudocolored images represent luciferase activity (photons/sec/cm²/sr) with radiance scales shown on the right.

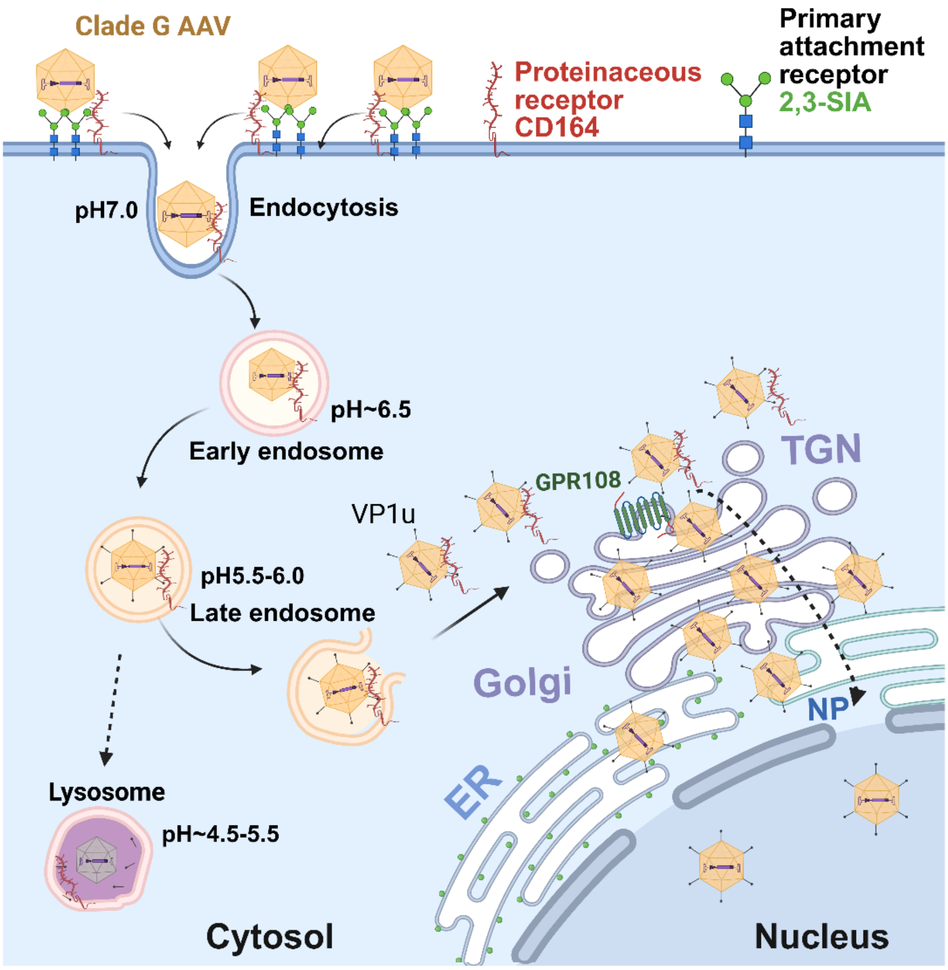

Fig. S12. A model of CD164 in the entry and intracellular trafficking of Clade G AAV.

Clade G AAV binds to the primary attachment receptor O-linked α-2,3-linked sialic acid (2,3-SIA) and the proteinaceous entry receptor CD164 on the cell surface. Following endocytosis at physiological pH (~7.0), the virus traffics through early endosomes (pH ~6.5) and late endosomes (pH ~5.5–6.0) where the VP1u is externalized from the capsid interior to the surface. A subset of virions evade lysosomal degradation and is routed through the trans-Golgi network (TGN), where GPR108 likely facilitates further trafficking of the virus through the Golgi apparatus and endoplasmic reticulum (ER) toward the nuclear membrane, where the nuclear pore (NP) enables the virus to enter the nucleus. Created by Biorender.

S1 References

1. **Pillay, S., N. L. Meyer, A. S. Puschnik, O. Davulcu, J. Diep, Y. Ishikawa, L. T. Jae, J. E. Wosen, C. M. Nagamine, M. S. Chapman, and J. E. Carette**. 2016. An essential receptor for adeno-associated virus infection. Nature. **530**:108-112.

2. **Zabner, J., P. Karp, M. Seiler, S. L. Phillips, C. J. Mitchell, M. Saavedra, M. Welsh, and A. J. Klingelhutz**. 2003. Development of cystic fibrosis and noncystic fibrosis airway cell lines. Am.J.Physiol Lung Cell Mol.Physiol. **284**:L844-L854.

3. **Yan, Z., X. Deng, and J. Qiu**. 2020. Human Bocavirus 1 Infection of Well-Differentiated Human Airway Epithelium. Curr.Protoc.Microbiol. **58**:e107.

4. **Qiu, J., R. Nayak, G. E. Tullis, and D. J. Pintel**. 2002. Characterization of the transcription profile of adeno-associated virus type 5 reveals a number of unique features compared to previously characterized adeno-associated viruses. J.Virol. **76**:12435-12447.

5. **Wang, Z., F. Cheng, J. F. Engelhardt, Z. Yan, and J. Qiu**. 2018. Development of a Novel Recombinant Adeno-Associated Virus Production System Using Human Bocavirus 1 Helper Genes. Mol.Ther.Methods Clin.Dev. **11**:40-51.

6. **Havlik, L. P., A. Das, M. Mietzsch, D. K. Oh, J. Ark, R. McKenna, M. Agbandje-McKenna, and A. Asokan**. 2021. Receptor Switching in Newly Evolved Adeno-associated Viruses. J.Virol. **95**:e0058721.

7. **Yang, R., N. T. Tran, T. Chen, M. Cui, Y. Wang, T. Sharma, Y. Liu, J. Zhang, X. Yuan, D. Zhang, C. Chen, Z. Shi, L. Wang, Y. Dai, H. Zaidi, J. Liang, M. Chen, D. Jaijyan, H. Hu, B. Wang, C. Xu, W. Hu, G. Gao, D. Yu, P. W. L. Tai, and Q. Wang**. 2025. AAVone: A cost-effective, single-plasmid solution for efficient AAV production with reduced DNA impurities. Mol.Ther.Nucleic Acids. **36**:102563.

8. **Zhang, X., S. Hao, Z. Feng, K. Ning, K. C. Aksu, S. McFarlin, D. Richart, F. Cheng, A. Zhang-Chen, R. McFarlane, Z. Yan, and J. Qiu**. 2025. Identification of SLC35A1 as an essential host factor for the transduction of multi-serotype recombinant adeno-associated virus (AAV) vectors. MBio. **16**:e0326824.

9. **Ning, K., C. A. Kuz, F. Cheng, Z. Feng, Z. Yan, and J. Qiu**. 2023. Adeno-Associated Virus Monoinfection Induces a DNA Damage Response and DNA Repair That Contributes to Viral DNA Replication. MBio. **14**:e0352822.

10. **Yan, Z., N. W. Keiser, Y. Song, X. Deng, F. Cheng, J. Qiu, and J. F. Engelhardt**. 2013. A novel chimeric adenoassociated virus 2/human bocavirus 1 parvovirus vector efficiently transduces human airway epithelia. Mol.Ther. **21**:2181-2194.

11. **Sanson, K. R., R. E. Hanna, M. Hegde, K. F. Donovan, C. Strand, M. E. Sullender, E. W. Vaimberg, A. Goodale, D. E. Root, F. Piccioni, and J. G. Doench**. 2018.

Optimized libraries for CRISPR-Cas9 genetic screens with multiple modalities. Nat.Commun. **9**:5416-07901.

12. **Li, W., H. Xu, T. Xiao, L. Cong, M. I. Love, F. Zhang, R. A. Irizarry, J. S. Liu, M. Brown, and X. S. Liu**. 2014. MAGeCK enables robust identification of essential genes from genome-scale CRISPR/Cas9 knockout screens. Genome Biol. **15**:554-0554.

13. **Thomas, P. D., M. J. Campbell, A. Kejariwal, H. Mi, B. Karlak, R. Daverman, K. Diemer, A. Muruganujan, and A. Narechania**. 2003. PANTHER: a library of protein families and subfamilies indexed by function. Genome Res. **13**:2129-2141.

14. **Mi, H., A. Muruganujan, D. Ebert, X. Huang, and P. D. Thomas**. 2019. PANTHER version 14: more genomes, a new PANTHER GO-slim and improvements in enrichment analysis tools. Nucleic Acids Res. **47**:D419-D426.

15. **Cheng, F., A. Y. Chen, S. M. Best, M. E. Bloom, D. Pintel, and J. Qiu**. 2009. The capsid proteins of Aleutian mink disease virus (AMDV) activate caspases and are specifically cleaved during infection. J.Virol. **84**:2687-2696.

16. **Ning, K., Z. Wang, F. Cheng, Z. Yan, and J. Qiu**. 2022. The small nonstructural protein NP1 of human bocavirus 1 directly interacts with Ku70 and RPA70 and facilitates viral DNA replication. PLoS.Pathog. **18**:e1010578.
